# Tumor NOS2 and COX2 Spatial Juxtaposition with CD8^+^ T Cells Promote Metastatic and Cancer Stem Cell Niches that Lead to Poor Outcome in ER^−^ Breast Cancer

**DOI:** 10.1158/2767-9764.CRC-24-0235

**Published:** 2024-10-23

**Authors:** Lisa A. Ridnour, William F. Heinz, Robert Y.S. Cheng, Adelaide L. Wink, Noemi Kedei, Milind Pore, Fatima Imtiaz, Elise L. Femino, Ana L. Gonzalez, Leandro L. Coutinho, Rebecca L. Moffat, Donna Butcher, Elijah F. Edmondson, Xiaoxian Li, Maria Cristina Rangel, Robert J. Kinders, Jens Rittscher, Stanley Lipkowitz, Stephen T.C. Wong, Stephen K. Anderson, Daniel W. McVicar, Sharon A. Glynn, Timothy R. Billiar, Jenny C. Chang, Stephen M. Hewitt, Stefan Ambs, Stephen J. Lockett, David A. Wink

**Affiliations:** 1 Cancer Innovation Laboratory, Center for Cancer Research, National Cancer Institute, National Institutes of Health, Frederick, Maryland.; 2 Optical Microscopy and Analysis Laboratory, Cancer Research Technology Program, Frederick National Laboratory for Cancer Research, Frederick, Maryland.; 3 Collaborative Protein Technology Resource (CPTR) Nanoscale Protein Analysis, Office of Science and Technology Resources, Center for Cancer Research, National Cancer Institute, National Institutes of Health, Bethesda, Maryland.; 4 Imaging Mass Cytometry, Frederick National Laboratory for Cancer Research, Frederick, Maryland.; 5 Optical Microscopy and Analysis Laboratory, Office of Science and Technology Resources, Center for Cancer Research, National Cancer Institute, National Institutes of Health, Frederick, Maryland.; 6 Molecular Histopathology Laboratory, Frederick National Laboratory for Cancer Research, National Cancer Institute, Frederick, Maryland.; 7 Department of Pathology and Laboratory Medicine, Emory University, Atlanta, Georgia.; 8 Center for Translational Research in Oncology, ICESP/HC, Faculdade de Medicina da Universidade de São Paulo and Comprehensive Center for Precision Oncology, Universidade de São Paulo, São Paulo, Brazil.; 9 Division of Cancer Treatment and Diagnosis, National Cancer Institute, National Institutes of Health, Frederick, Maryland.; 10 Institute of Biomedical Engineering, Big Data Institute, Ludwig Oxford Branch, University of Oxford, Oxford, United Kingdom.; 11 Women’s Malignancies Branch, Center for Cancer Research, National Cancer Institute, National Institutes of Health, Bethesda, Maryland.; 12 Houston Methodist Neal Cancer Center, Weill Cornell Medical College, Houston Methodist Hospital, Houston, Texas.; 13 Basic Science Program, Frederick National Laboratory for Cancer Research, Frederick, Maryland.; 14 Discipline of Pathology, Lambe Institute for Translational Research, School of Medicine, University of Galway, Galway, Ireland.; 15 Department of Surgery, University of Pittsburgh Medical Center, Pittsburgh, Pennsylvania.; 16 Laboratory of Pathology Center for Cancer Research, National Cancer Institute, National Institutes of Health, Bethesda, Maryland.; 17 Laboratory of Human Carcinogenesis, Center for Cancer Research, National Cancer Institute, National Institutes of Health, Bethesda, Maryland.

## Abstract

Estrogen receptor–negative breast cancer is an aggressive subtype with limited therapeutic options. Elevated nitric oxide synthase 2 (NOS2) and COX2 mediate immunosuppression and poor survival in these tumors. Therefore, the influence of tumor NOS2/COX2 on immune architecture was examined in 16 African American and five Caucasian estrogen receptor–negative tumors. Elevated tumor NOS2/COX2 limited CD8^+^ T-cell infiltration at 5-year survival. Distinct CD8^+/−^NOS2^+/−^COX2^+/−^ phenotypes defining metastatic and cancer stem cell niches and immune desert regions were identified. These results were supported by an unbiased, unsupervised nonlinear dimensionality reduction technique, Uniform Manifold Approximation and Projection for Dimension Reduction, incorporating spatial relations between cells and validated in a separate gene expression cohort using NOS2/CD8 and COX2/CD8 ratios. Additionally, elongated tumor cells were specifically in CD8^−^NOS2^+^COX2^+^ regions, suggesting metastatic hot spots. This work demonstrates predictive power of spatial analyses of CD8/NOS2/COX2 architecture and supports the use of clinically available NOS2/COX2 inhibitors for improved survival in patients with these aggressive tumors.

**Significance:**

This work identifies CD8^−^NOS2^+^COX2^+^ and CD8^−^NOS2^−^COX2^+^ unique cellular neighborhoods that drive the tumor immune spatial architecture of CD8^+^ T cells predictive of clinical outcome and can be targeted with clinically available NOS inhibitors and NSAIDs.

## Introduction

Breast cancer remains a leading malignancy in women. It is a heterogeneous disease discerned by hormonal and HER2 status (1). Although many treatment options are available for estrogen receptor–positive (ER^+^) tumors, ER-negative (ER^−^) and triple negative breast cancer (TNBC) subtypes are the most clinically challenging malignancies due to limited treatment options (1). Recently, elevated tumor nitric oxide synthase 2 (NOS2) and COX2 coexpression has shown strong predictive power, in which their elevated expression correlates with reduced survival in ER^−^ but not ER^+^ breast cancer (2). *In vitro* studies have elucidated an intercellular feed forward signaling loop between the NOS2 and COX2 enzymes, in which NO produced by NOS2 activates COX2 to produce prostaglandin E2 (PGE2), which in turn activates NOS2. This loop leads to increased chemoresistance, cancer stemness, metastasis, and immunosuppression (2–5). Importantly, immunosuppression is a key mechanism limiting cancer treatment efficacy (6). Many studies have shown that COX2-derived PGE2 and NOS2-derived NO promote immunosuppression in part by limiting activated T effector cells through increased ARG1, IL10, and TGFβ (7–10). In contrast, NOS2/COX2 blockade augments cytolytic T cells, mature B cells, and neutrophils associated with resistance to 4T1 tumor rechallenge in 4T1 tumor-bearing mice (11). Notably, these results are consistent with a recent phase I/II clinical trial demonstrating remodeling of the tumor immune microenvironment that involved increased B cells and neutrophils as well as improved overall survival and complete pathologic responses in patients with chemoresistant TNBC treated with Taxol and the NOS inhibitor, L-NMMA (12).

Recent studies have shown that the spatial localization of CD8^+^ T cells is predictive of TNBC clinical outcomes, in which CD8^+^ T-cell penetration into the tumor core (immune inflamed) correlated with proinflammatory immune response and predicted improved survival (13). In contrast, tumors with stroma-, margin-restricted, or abated CD8^+^ T cells (immune cold or immune desert, respectively) are immunosuppressed and predictive of poor clinical outcomes (13). As previously shown by us, elevated tumor COX2 expression correlates with abated CD8^+^ T-cell infiltration into breast tumors, whereas stroma-restricted CD8^+^ T cells correlate with both increased tumor NOS2 expression and tumor budding or satellitosis (14). Thus, we hypothesized that spatial relationships exist between tumor NOS2 and COX2 expression and infiltrating CD8^+^ T cells that could influence clinical outcomes by promoting immunosuppression and increased disease aggressiveness.

Given that tumor NOS2 and COX2 expression is antagonistic against CD8^+^ T-cell function, we used NOS2/CD8 and COX2/CD8 ratios to define specific cellular phenotypes, which revealed significant spatial relationships with respect to patients that survived (alive) versus those who succumbed to disease (deceased) at 5 years postdiagnosis in 21 patients with ER^−^ breast cancer (16 African American and five European American). CD8^+^NOS2^−^COX2^−^ phenotypes defined fully inflamed tumors with augmented CD8^+^ T-cell infiltration in tumors from surviving patients at 5 years. In contrast, elevated NOS2 and COX2 expression in tumors from deceased patients exhibited inflamed CD8^+^NOS2^+^COX2^+^ regions with stroma-restricted CD8^+^ T cells and CD8^−^NOS2^−^COX2^+^ immune desert regions with abated CD8^+^ T-cell penetration. Also, spatial analysis identified cells positive for the cancer stem cell (CSC) markers, CD44v6 and EpCAM, within these cellular neighborhoods that could contribute to stemness, drug resistance, and metastasis. Together, these spatially defined NOS2 and COX2 expression patterns shape the tumor immune microenvironment and identify cellular neighborhoods that promote cancer disease progression.

## Materials and Methods

### Tissue collection and IHC analysis of patient tumor sections

A retrospective study based upon a historical collection of tumor specimens obtained from patients with breast cancer recruited at the University of Maryland (UMD) Medical Center, the Baltimore Veterans Affairs Medical Center, Union Memorial Hospital, Mercy Medical Center, and the Sinai Hospital in Baltimore between 1993 and 2003 is reported. Written informed consent was obtained from all patients. Clinical and pathologic information was obtained from medical records and pathology reports and associated with unique patient identifier numbers. Disease staging was performed according to the tumor–node–metastasis system of the American Joint Committee on Cancer/Union Internationale Contre le Cancer (15). The Nottingham system was used to determine the tumor grade (16). Studies were conducted in accordance with recognized ethical NIH guidelines followed by the Declaration of Helsinki and performed after approval by an institutional review board (IRB) and in accordance with an assurance filed with and approved by the US Department of Health and Human Services. The collection of tumor specimens, survey data, and clinical and pathologic information (UMD protocol no. 0298229) was reviewed and approved by the UMD IRB for the participating institutions. The research was also reviewed and approved by the NIH Office of Human Subjects Research (OHSR no. 2248). IRB approval of this protocol was obtained at all institutions (Veterans Affairs Medical Center, Union Memorial Hospital, Mercy Medical Center, and Sinai Hospital). There was no linkage to personal identifiers for these patients, and attrition information was not available. Each tumor sample was identified by an accession number for blinding purposes. Breast tumor NOS2 and COX2 expression was analyzed previously by IHC using 1:250 diluted NOS2 antibody and 1:50 diluted COX2 antibody [no. 610328 (RRID: AB_397718) and 610204 (RRID: AB_397603), respectively, BD Biosciences] and scored by a pathologist (4, 17). For NOS2 staining, a combination score of intensity and distribution was used to categorize the IHC NOS2 stains where intensity received a score of 0 to 3 if the staining was negative, weak, moderate, or strong. The NOS2 distribution received scores of 0 to 4 for distributions <10%, 10% to 30%, >30 to 50%, >50 to 80%, and >80% positive cells (4). For COX2 staining, scores of negative to weak (1–2) or moderate to strong (3–4) were categorized as low or high, respectively (17). Herein, the tumor immune microenvironment was examined in NOS2_Hi_/COX2_HI_ (*n* = 11) versus NOS2_Lo_/COX2_Lo_ (*n* = 10) expressing ER^−^ tumors, which included tumors with TNBC (16) and HER2-positive (5) status and correlated with 5-year survival. Of the 21 ER^−^ samples examined, nine patients received presurgery/neoadjuvant treatment prior to tumor resection. However, at the time of tumor resection, which was performed after a recovery period, the influence of neoadjuvant therapy should have disappeared. We examined tumors that were either NOS2Hi/COX2Hi (11) or NOS2Lo/COX2Lo (10) in tumor expression. Some tumors were NOS2Hi/COX2Lo or NOS2Lo/COX2Hi, and these samples were excluded. In addition, some tumors were excluded based upon disease-specific survival at 5 years postdiagnosis; some patients died of unknown or other causes. Several blocks were no longer available due to usage/tissue exhaustion and could not be examined. Specific medical review for follow-up with regard to recurrence was not available; the National Death Index was searched in 2006, 2008, 2010, and 2020 for survival follow-up. Since the 5-year mark, four women have succumbed with malignant neoplasm of breast at 24.3, 10.4, 8.4, and 6.5 years postdiagnosis or surgery. Information of active disease in surviving patients at the 5-year mark was not available.

The influence of tumor NOS2 and COX2 expression on 5-year disease-specific survival is reported. NOS2 and COX2 expressions were analyzed by fluorescent staining performed on the Leica Biosystems BOND RX Autostainer XL ST5010 using the BOND Polymer Refine Kit (Leica Biosystems DS9800), with omission of the Post Primary Block reagent, 3, 3′-diaminobenzidine (DAB), and hematoxylin. After antigen retrieval with EDTA (BOND Epitope Retrieval 2), sections were incubated for 30 minutes with COX2 [Cell Signaling Technology, no. 12282 (RRID: AB_2571726), 1:100], followed by the polymer reagent and Opal Fluorophore 520 (Akoya Biosciences). The COX2 antibody complex was stripped by heating with BOND Epitope Retrieval 2. Sections were then incubated for 30 minutes with NOS2 antibody [Abcam no. ab15323 (RRID: AB_301857), 1:50], followed by the polymer reagent and Opal Fluorophore 690. The NOS2 antibody complex was stripped by heating with BOND Epitope Retrieval 2 and then stained with CD8 [Abcam no. 101500 (RRID: AB_10710024), 1:100] or IFNγ [Abcam no. 231036 (RRID: AB_2941995), 1:200], followed by the polymer reagent and Opal Fluorophore 570. Sections were stained with 4′,6-diamidino-2-phenylindole (DAPI) and coverslipped with ProLong Gold AntiFade Reagent (Invitrogen). Images were captured using the Aperio ScanScope FL whole slide scanner (Leica Biosystems). The original IHC previously reported (4, 17) and fluorescent NOS2/COX2 staining results were generally consistent.

Formalin-fixed paraffin-embedded tissue sectioned at 4 μm and mounted on SuperFrost Plus slides were stained with a FixVUE Immuno-8 Kit [RRID: AB_3251507; formerly referred to as UltiMapper kits (Ultivue Inc), CD8, NOS2, COX2, CKSOX10, and IFNγ cocktail] using the antibody-conjugated DNA-barcoded multiplexed immunofluorescence (mIF) method (1). These kits include the required buffers and reagents to run the assays: antibody diluent, preamplification mix, amplification enzyme and buffer, fluorescent probes and corresponding buffer, and nuclear counterstain reagent. Hematoxylin and eosin and mIF staining was performed using the Leica Biosystems BOND RX Autostainer. Before performing the mIF staining, formalin-fixed paraffin-embedded tissue sections were baked vertically at 60°C to 65°C for 30 minutes to remove excess paraffin prior to loading on the BOND RX. The BOND RX was used to stain the slides with the recommended FixVUE (UltiMapper) protocol. During assay setup, the reagents from the kit were prepared and loaded onto the Autostainer in Leica Titration containers. Solutions for epitope retrieval (ER2, Leica Biosystems, cat. # AR9640), BOND Wash (Leica Biosystems, cat. # AR9590), along with all other BOND RX bulk reagents, were purchased from Leica Biosystems. During this assay, the sample was first incubated with a mixture of all four antibody conjugates; next, the DNA barcodes of each target were simultaneously amplified to improve the sensitivity of the assay. Fluorescent probes conjugated with complementary DNA barcodes were then added to the sample to bind and label the targets; next, a gentle signal removal step was used to remove the fluorescent probes of the markers. The stained slides were mounted in ProLong Gold AntiFade Mountant (Thermo Fisher Scientific, cat. # P36965) and coverslipped (Fisherbrand Cover Glass 22 × 40 mm, #1.5). Digital immunofluorescence images were scanned at 20× magnification. Images were coregistered and stacked with Ultivue UltiStacker software. The digital images were then analyzed using HALO image analysis platform (18).

### Genome Expression Omnibus

The GSE37751 breast cancer data were obtained from the Genome Expression Omnibus public data repository (https://www.ncbi.nlm.nih.gov/geo/info/download.html; RRID: SCR_005012). The R software (version 4.2) was used to extract gene expression data from ER^−^ samples for subsequent analysis. Briefly, NOS2, COX2, and CD8A gene expression and associated survival data were extracted and processed together. The data set was divided into two subsets by the median NOS2 and COX2 expression value (high vs. low). High (red) and low (black) NOS2/CD8A and COX2/CD8A ratios dichotomized at the median were calculated. The associated survival was exported to Prism (v10), and probability of survival was plotted. The *P* values were determined using log-rank (Mantel–Cox) test, and HRs were calculated using the Mantel–Haenszel test.

#### *In vivo* studies

Animal care was provided at the NCI-Frederick Animal Facility according to procedures outlined in the Guide for Care and Use of Laboratory Animals. Our facility is accredited by the Association for Accreditation of Laboratory Animal Care International and follows the Public Health Service Policy for the Care and Use of Laboratory Animals. Female BALB/c mice (RRID: MGI:2683685) obtained from the Frederick Cancer Research and Development Center Animal Production Area were used for the *in vivo* studies and housed five per cage. Eight to ten-week-old female wild-type and Nos2^−^ BALB/c mice were shaved a day prior to tumor injection and then were injected subcutaneously into the fourth mammary fat pad with 200,000 4T1 TNBC cells obtained from ATCC (RRID: CVCL_0125). The cells were authenticated by the vendor. Upon receipt, the cells were expanded and stored at passage (p.) 8 and not used beyond p.30. Tumor measurements began 1 week after tumor cell injection, using a Vernier caliper and calculated in cubic millimeter volumes according to the following equation:

$$\left[{\left(\text{short}\;\text{diameter}\right)}^{2}\times \;\text{long}\;\text{diameter}\right]/2.$$

Upon reaching tumor size of 100 mm^3^, tumor-bearing mice were divided into groups and treatment with 30 mg/L indomethacin in drinking water was initiated and mice were treated for the next 7 days. The water was changed every Monday, Wednesday, and Friday for the duration of the experiment. Mice were euthanized, and tumor tissues were harvested and flash frozen. Ten-micrometer tissue sections were mounted on coverslips for CODEX imaging.

### CODEX analysis

The CODEX protocol was performed according to Akoya User Manual, revision B.0. Square (22 × 22 mm) glass coverslips (72204-10, Electron Microscopy Sciences) were pretreated with L-Lysine [#P8920, Sigma (RRID: SCR_008988)] overnight at room temperature. Coverslips were rinsed in distilled water, dried, and stored at room temperature. Fresh frozen tissue blocks were sectioned (10 μm) on treated coverslips and stored in a coverslip storage box (Qintay, LLC) at −80°C until further use. CODEX reagents and instrumentation were purchased from Akoya Biosciences. Antibodies labeled for CODEX included CD279 (RRID: AB_313418), CD86 (RRID: AB_47368), Ki67 (RRID: AB_2895046), E-cadherin (RRID: AB_2291471), CD19 (RRID: AB_3254289), PIMO (RRID: AB_3254477), CD31 (RRID: AB_3254603), CD49f (RRID: AB_3254703), vimentin (RRID: AB_10695459), F4-80 (RRID: AB_3271523), αSMA (RRID: AB_2572996), CD44v6 (RRID: AB_10597738), Ly6C (RRID: AB_1134213), NOS2 (RRID: AB_3271526), CD206 (RRID: AB_2538349), CD25 (RRID: AB_312856), CD11c (RRID: AB_3271529), CD274 (RRID: AB_467784), CD44 (RRID: AB_3271530), CD24 (RRID: AB_3271531), MHCII (RRID: AB_3271532), CD3 (RRID: AB_3271528), CD90 (RRID: AB_3271533), CD5 (RRID: AB_3271534), CD71 (RRID: AB_3271535), CD45 (RRID: AB_3271536), CD4 (RRID: AB_3271537), CD169 (RRID: AB_3271538), CD38 (RRID: AB_3271539), CD8a (RRID: AB_3271540), Ly6G (RRID: AB_3271541), and CD11b (RRID: AB_3271542). Tissue sections were stained with an antibody cocktail consisting of 0.5 to 1 μL of each antibody per tissue. CODEX assays were performed according to the manufacturer’s recommendations. Fluorescent oligonucleotide plates were prepared in black 96-well plates for image acquisition. Each CODEX cycle contains four fluorescent channels (three for antibody visualization and one for nuclear stain). For each cycle, up to three fluorescent oligonucleotides (5 μL each) were added to a final volume of 250 μL of plate buffer (containing Hoechst nuclear stain). For blank (empty) cycles, 5 μL of plate buffer was substituted for fluorescent oligonucleotides. Plates were sealed and kept at 4°C until use. For imaging, the CODEX coverslip was mounted onto a custom-designed plate holder and securely tightened onto the stage of a Keyence BZ-X810 inverted fluorescence microscope. Cycles of hybridization, buffer exchange, image acquisition, and stripping were then performed using an Akoya CODEX instrument. Briefly, that instrument performs hybridization of the fluorescent oligonucleotides in a hybridization buffer, imaging of tissues in CODEX buffer, and stripping of fluorescent oligonucleotides in the stripping buffer. CODEX multicycle automated tumor imaging was performed using a CFI Plan Apo 20×/0.75 objective (Nikon). The multipoint function of the BZ-X viewer software (BZ-X ver. 1.3.2, Keyence) was manually programmed to align with the center of each tumor and set to 10 Z stacks. Nuclear stain (DAPI, 1:600 final concentration) was imaged in each cycle at an optimized exposure time of roughly 10 ms. The respective channels were imaged in the automated run using optimized exposure times. Raw TIFF images produced during image acquisition were processed using the CODEX image processer. The processer concatenates Z-stack images, performs drift compensation based on alignment of nuclear stain across images, and removes the out-of-focus light using the Microvolution deconvolution algorithm (Microvolution). The processer also corrects for nonuniform illumination and subtracts the background and artefacts using blank imaging cycles without fluorescent oligonucleotides. The output of this image processing was tiled images corresponding to all fluorescence channels and imaging cycles that were then visualized and analyzed using HALO software (Version 3.3.2541.383, Indica Labs Inc.). Segmentation of cells was performed using the nuclear channel, and the cell cytoplasm was defined as a fixed width ring around each nucleus. Nuclear segmentation settings were optimized by visual verification of segmentation performance on random subsets of cells aiming to minimize the number of over segmentations, under segmentation, detected artefacts, and missed cells. Cell-type annotation and differential marker analysis cell populations were gated as follows. Tissues were annotated to exclude the edge effect. All nucleated cells were first identified by positive nuclear signals. Cell phenotypes were defined as CD44v6^+^CD45^−^ based upon biomarker expression as judged by expert visual inspection.

### Spatial Uniform Manifold Approximation and Projection for Dimension Reduction and neighborhood analysis

The spatial Uniform Manifold Approximation and Projection for Dimension Reduction (S-UMAP) and neighborhood analysis used here extends the analysis previously described by Giraldo and colleagues (19). Using NOS2 moderate and strong signal intensities, COX2 moderate signal intensities, and CD8 phenotypes (eight total phenotypes), each cell was analyzed and a phenotype density census, or neighborhood profile, was calculated using all nearby cells within specific distance ranges of 0 to 25, 25 to 50, 50 to 100, 100 to 150, and 150 to 200 μm. The resulting single cell neighborhood profile is a 40-value descriptor of that cell’s neighborhood. The neighborhood profiles of all cells in all samples (*N* = 1,263,845 cells total) were dimensionally reduced by a UMAP and plotted as a 2D histogram. The 2D cell histograms of tumors from deceased and alive patients comprising the S-UMAP were plotted, and the difference between the deceased and alive histograms was plotted as a log-ratio. The S-UMAP difference thresholds identified bins that were more prevalent in deceased versus alive patients (log-ratio thresholds of 0.01 and −0.01, respectively). Clusters of similar neighborhoods in these sets were identified in the 2D representation by adaptive k-means clustering. The optimal number of clusters for deceased and alive cell sets was selected by minimizing the Davies–Bouldin criterion, and the K-means clustering identified five clusters in the S-UMAP (A1 and A2, for alive; D1, D2, and D3 for deceased). Average neighborhoods of these clusters were calculated based on selected cells (e.g., cells belonging to a particular cluster). Ratios of clusters were calculated on a per-phenotype basis. Comparisons between cluster populations were based on 5-year outcome. Analyses were performed using MATLAB custom software and the UMAP library (https://www.mathworks.com/matlabcentral/fileexchange/71902), (MATLAB Central File Exchange RRID: SCR_001622]; ref. 20). Neighborhood profiles are plotted as mean ± SEM.

### Gene expression

Cells were seeded at 1 × 10^6^ cells per 60 mm Petri dish and incubated overnight. Then, cells were serum-starved in phenol red–free, serum-free RPMI supplemented with 1 mmol/L L-arginine (Sigma, Cas# 74-79-3) for 24 hours. Following starvation, the cells were treated with a cytokine mixture (100 U/mL IFNγ, 10 ng/mL TNFα, and 10 ng/mL IL1β) for 48 hours in phenol red–free RPMI containing 1 mmol/L L-arginine (Sigma, Cas# 74-79-3) and 10% FBS. RNA was extracted from the samples using TRIzol Reagent (Invitrogen) according to the manufacturer’s protocol, and RNA concentration was measured using a NanoDrop ND-1000 (Thermo Fisher Scientific Inc.). RNA was then converted to cDNA using EcoDry Premix (Takara Bio Inc). qRT-PCR was performed with SensiFAST Sybr Hi-Rox mix (Bioline, Cat. # BIO-92020) on a QuantStudio 3 Real-Time PCR System (Thermo Fisher Scientific, Cat. # A28567). GAPDH was used as a housekeeping gene for normalization of target gene expression. Gene expression analysis was conducted using oligonucleotide primers specific to the target genes. The primer sequences used were as follows:

NOS2 (R) ATC​TGG​AGG​GGT​AGG​CTT​GT, (F) CCA​TAA​GGC​CAA​AGG​GAT​TT; COX2 (R) AAA​ATT​CCG​GTG​TTG​AGC​AG, (F) TGA​GTG​TGG​GAT​TTG​ACC​AG; EpCAM (R) AAG​ATG​TCT​TCG​TCC​CAC​GC, (F) GCT​GGA​ATT​GTT​GTG​CTG​GT.

### NO measurement

NO levels were measured using the diaminonaphthalene (DAN) fluorescence assay for nitrite. Cells were seeded at a density of 4 × 10^4^ cells per well in clear flat-bottom 96-well plates and incubated overnight. Following incubation, the cells were serum-starved in phenol red–free, serum-free RPMI supplemented with 1 mmol/L L-arginine (Sigma, Cas# 74-79-3) for 24 hours. Subsequently, the cells were treated with a cytokine mixture (100 U/mL IFNγ and 10 ng/mL TNFα) for 48 hours in phenol red–free RPMI containing 1 mmol/L L-arginine (Sigma, Cas# 74-79-3), 10% FBS, 100 μmol/L 2,3-DAN (Fluka, Cat. # 33187), and 2 μmol/L carboxy-PTIO sodium salt (NO trap; Calbiochem, Cat. # 217386). After treatment, 150 μL of the supernatant was collected, transferred to a clear flat-bottom 96-well plate (Corning, Cat. # 3596), and centrifuged at 2,000 rpm for 5 minutes to remove cellular debris. Then, 100 μL of the supernatant was transferred to a black/clear 96-well plate (Thermo Fisher Scientific, Cat. # 165305), and DAN fluorescence intensity was measured using a SpectraMax i3x (Molecular Devices) with the following settings: Ex: 375 nm/Em: 425 nm; bandwidth, Ex: 15 nm/Em: 15 nm. Due to DAN’s photosensitivity, all measurements were conducted in the dark. Background fluorescence was subtracted, and the data were reported as ΔRFU.

### Statistical analysis

A power analysis was not conducted due to the exploratory nature of the research. We used robust statistical techniques to ensure the validity and reliability of our findings. Experiments were assayed in triplicate unless otherwise stated. Unpaired student *t* test was employed to assess statistical significance using the GraphPad Prism software (version 9; RRID: SCR_002798). Image analyses are reported as mean ± SEM, and *t* tests with Welch’s or Mann–Whitney correction were used when appropriate to determine significance. Linear analyses and Pearson’s correlations were also conducted to determine significant correlations between protein expressions using GraphPad Prism software. Significance is reported as ^∗^, *P* ≤ 0.05; ^∗∗^, *P* ≤ 0.01; ^∗∗∗^, *P* ≤ 0.001; and ^∗∗∗∗^, *P* ≤ 0.0001. Single cell correlation analyses were conducted in RStudio using the corrplot (0.92) in R (4.2.1).

### Data availability

RNA sequencing data will be made available upon request.

## Results

### Tumor NOS2/COX2 expression and survival

**Figure 1:**
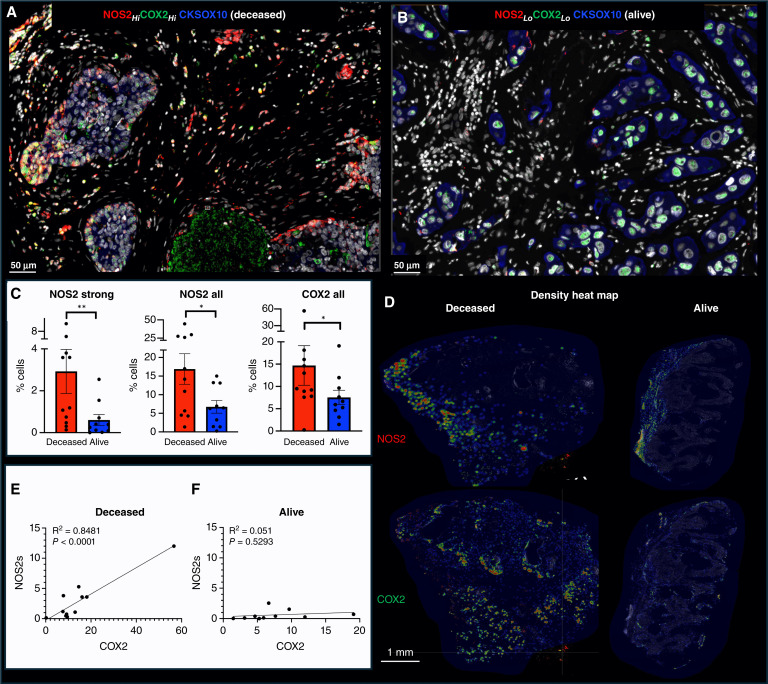
Spatial analysis of tumor NOS2/COX2 expression with respect to survival. Spatial landscape of NOS2 (red) and COX2 (green) with DAPI (white), the tumor marker CKSOX10 (blue) for (**A**) deceased and (**B**) alive patients at 5-year survival. **C,** Quantification of NOS2/COX2 tumor expression at the single cell level, with each dot representing a tumor sample (also shown in Supplementary Fig. S2). **D,** Heat density maps of tumor NOS2/COX2 expression in deceased and alive patient tumors. **E,** Significant Pearson’s correlation between tumor NOS2 and COX2 expression *R*^2^ = 0.8481 and *P* < 0.0001 in tumors from deceased patients but not alive patient samples (**F**). Mann–Whitney *, *P* < 0.05; **, *P ≤ *0.01.

Previously, pathologic IHC scoring revealed the predictive power of elevated tumor NOS2 and COX2 expression relative to poor survival at 5 years postdiagnosis in ER^−^ but not ER^+^ breast cancer (2, 4, 17). Tumor NOS2 expression was moderately to strongly expressed in 70% of all (ER^+^ and ER^−^) tumors, whereas only a few tumors (10%–20%) showed marked NOS2 expression in infiltrating CD11b^+^ immune cells, implicating the tumor epithelium as the major source of NOS2-derived NO in breast tumors (4). Importantly, breast cancer cells can be induced to express NOS2 and COX2 expression (Supplementary Fig. S1A) and generate NO (Supplementary Fig. S1B; refs. 2, 3, 21), and exogenous NO promotes migration and invasion of these aggressive cells (3, 4, 14, 21). Among all samples examined in this study, NOS2 and COX2 proteins were elevated in adjacent normal breast tissue in only one sample, and COX2 protein alone was elevated in adjacent normal breast tissue of a second sample. Both tissues had high tumor NOS2 and COX2 expression and both patients succumbed to disease within 3 years of diagnosis. All other samples were either low or negative for NOS2 and COX2 protein in adjacent normal breast tissue. Herein, we extend these findings using mIF imaging, which facilitates the spatial localization of individual cells along with their combination of expressed targets. Spatial localization of cells was defined in the histologic context of the tissue as determined by annotations of viable tumor, necrotic tumor, and tumor stroma regions applied by a veterinary pathologist (EFE) on hematoxylin and eosin serial sections adjacent to those used for immunofluorescence of ER^−^ breast tumors (*n* = 21 including 16 TNBC and five HER2-positive) from a previously reported cohort (2, 4, 17). This methodology provides both quantitative and mechanistic descriptions of different cellular neighborhoods within the tumor microenvironment (TME) that correlate with disease progression and was applied to the cohort. Consistent with the original pathologist IHC scores, the average NOS2/COX2 fluorescence cell intensities were significantly higher in tumors from deceased versus alive patients at 5-year survival (Fig. 1A–C; refs. 4, 17). Quantitative analysis of tumor NOS2 and COX2 expressions revealed multiple levels of signal intensity for NOS2, but not COX2. Therefore, the NOS2 and COX2 levels were quantized per cell by classifying each cell by weak, moderate, or strong NOS2 and COX2 levels (Fig. 1C; Supplementary Fig. S2A–S2C; ref. 14). When stratifying for survival, the NOS2 and COX2 signal intensities in tumors from deceased versus alive patients demonstrated a higher fold-change (∼6×) in the strong signal intensities of NOS2 (NOS2s) compared with moderate or weak (∼2–3×) signal intensities (Supplementary Fig. S2A). In contrast, the fold-change for COX2 was similar (2–3×) across the three levels (Supplementary Fig. S2B). These results suggest that NOS2 fluorescence signal intensity could have higher predictive value than COX2, which is supported by NOS2 and COX2 HR of 6.19 and 2.79, respectively, as reported by Glynn and colleagues (4, 14).

Earlier studies identified NOS2 and COX2 feedforward signaling that supports many oncogenic pathways in ER^−^ breast cancer (2, 3). Moreover, a strong linear relationship between tumor NOS2 and COX2 expression in all patient tumors has been reported (14). Given that the flux of NOS2-derived NO depends on the local density of NO producing cells as well as the concentration, diffusion, and reaction kinetics (22), we used density heat map analyses (Fig. 1D) to assess tumor NOS2 and COX2 clustering in tumors from deceased versus alive patients. Visual differences in density heat maps revealed increased spatially distinct tumor NOS2 and COX2 clustering indicative of increased, spatially distinct regional NO and PGE2 flux in deceased patient tumors (Fig. 1D). Moreover, a robust linear correlation was observed between strong NOS2 signal versus COX2 signal intensities in tumors from deceased (Fig. 1E; *R*^2^ = 0.8481, *P* < 0.0001) but not alive patients (Fig. 1F; *R*^2^ = 0.051, *P* = 0.5293 F). These findings indicate that upregulated tumor NOS2 and COX2 expression at the single cell level is predictive of poor survival and is consistent with the previous NOS2 and COX2 IHC scoring, which showed strong predictive power of tumor NOS2 and COX2 expression in ER^−^ breast cancer (2, 4, 17).

### Predictive power of CD8^+^ T-cell infiltration in the context of tumor NOS2 and COX2 expression

**Figure 2:**
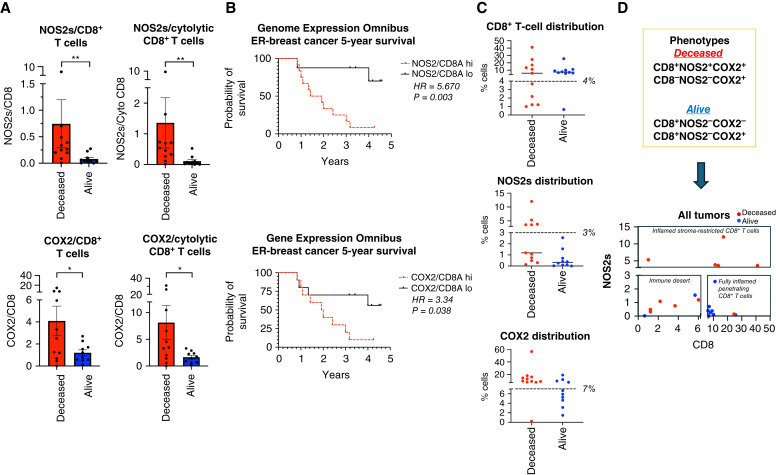
Analysis of tumor NOS2/COX2 expression with respect to CD8^+^ T cells. **A,** Ratio of NOS2s/CD8 and COX2/CD8 expression in total and CD3^+^CD8^+^PD1^−^ cytolytic T-cell populations. **B,** ER^−^ breast cancer at 5-year survival stratified for high vs. low NOS2/CD8 and COX2/CD8 ratio validation in GSE37751 breast cancer data from Genome Expression Omnibus public data repository (https://www.ncbi.nlm.nih.gov/geo/info/download.html). The R software (version 4.2) was used to extract gene expression data from ER^−^ samples for analysis of high (red) vs. low (black) NOS2/CD8a and COX2/CD8a ratios dichotomized at the median. The survival data were exported to Prism (version 9) and plotted for survival. HRs and *P* values were determined using Mantel–Haenszel and Gehan–Breslow–Wilcoxon test in Prism software. **C,** Phenotype % cell distributions in each tumor sample relative to survival. Each dot represents a tumor sample, and the dashed lines denote natural breaks in the data. **D,** Distribution plots of NOS2s vs. CD8^+^ T cells of all samples showing distinct regional clustering of immune deserts, fully inflamed penetrating CD8^+^ T cells, and inflamed stroma-restricted CD8^+^ T cells in tumors from deceased (red) vs. alive (blue) patients. *, *P* ≤ 0.05; **, *P* ≤ 0.01.

The increased presence of CD8^+^ T cells in the tumor core is predictive of improved TNBC survival, whereas their limited infiltration and/or stroma restriction correlates with poor clinical outcomes (13, 23, 24). Given that (i) the NOS2/COX2 products NO/PGE2 are antagonistic to T effector cell function, (ii) that tumor NOS2 and COX2 expression limited CD8^+^ T-cell penetration into the tumor core, and (iii) that potent immune responses potentiate therapeutic efficacies, we reasoned that tumor NOS2 and COX2 expression could impact clinical outcomes by effects on CD8^+^ T-cell penetration and function. Thus, we examined the influence of tumor NOS2 and COX2 expression on the CD8^+^ T-cell landscape in the ER^−^ breast cancer cohort. Significant differences in the total numbers of CD8^+^ T cells, CD8 T_*Eff*_/CD8 ratio, or other immune markers were not observed between tumors from deceased and alive patients (Supplementary Fig. S3A TNBC tumors). However, quantified NOS2s/CD8 and COX2/CD8 ratios were significantly elevated in tumors from deceased patients, for both total and cytolytic (CD8^+^PD1^−^) CD8^+^ T-cell populations (Fig. 2A). Mechanistically, PGE2 released from COX2-expressing tumor cells impairs the interaction of conventional dendritic cells (cDC1) with CD8^+^ T cells leading to decreased antigen presentation and recruitment of CD8^+^ T cells to the TME (25, 26). We explored this possibility in 4T1 tumor-bearing mice treated with the clinically available NSAID indomethacin (INDO) that targets COX2-expressing cells (11) and increases the PGE2 consumptive enzyme 15-PGDH (27). When compared with control untreated mice, bulk RNA sequencing analysis shows that INDO treatment led to increased expression of the cDC1 lineage determining factor IRF8, the c-type lectin-like activation receptor CLEC9a involved in antitumor immunity, chemokines CXCL9-11 that promote directional migration of immune cells, and IL27, which synergizes with IL12 to promote IFNγ production by CD4^+^, CD8^+^ T cells, and NKT cells (Supplementary Fig. S3B murine 4T1 tumors; refs. 26, 28).

The elevated NOS2/CD8 and COX2/CD8 relationships were validated in the Gene Expression Omnibus database inclusive for ER^−^ breast cancer subtype, which showed significant HRs of 5.67 and 3.34 at 5-year survival, for high versus low NOS2/CD8 and COX2/CD8 ratios, respectively, dichotomized at the median (Fig. 2B). Importantly, these HRs are consistent with those published earlier for tumor NOS2 (HR = 6.19) and COX2 (HR = 2.79) expressions (4, 17). These results suggest that elevated tumor NOS2 and COX2 expression can suppress cytolytic CD8^+^ T effector cell populations during TNBC disease progression.

To spatially explore the impact of these relationships on clinical outcomes, tumor NOS2/COX2 expression was assessed in conjunction with CD8^+^ T-cell counts (Fig. 2C). Significantly, barring one tumor from deceased and two tumors from alive patients, deceased patient tumors had mixed NOS2 and CD8^+^ T cell levels, which could be separated into both NOS2 and CD8 low immune desert regions and NOS2 high, stroma-restricted CD8^+^ T-cell inflamed regions (Fig. 2D). Alive patient tumors had concurrently both high CD8^+^ T-cell counts and low NOS2 levels (Fig. 2D). In addition, except for one, deceased patients were COX2 high, but COX2 levels were mixed in alive patients (Fig. 2C). This separation between surviving patients versus those who succumbed to disease not only demonstrates the predictive power of these biomarkers relative to survival but more importantly could serve to guide therapeutic options for patients with these aggressive tumors. Importantly, FDA-approved drugs that specifically target NOS2 and COX2 are clinically available, which could readily be combined with immunotherapeutics targeting inactivated CD8^+^ T cells. Indeed, a recent and successful phase I/II clinical trial using the pan-NOS inhibitor L-NMMA combined with docetaxel and low-dose aspirin showed complete or partial remission in patients with drug-resistant TNBC and locally advanced breast cancer who had otherwise exhausted treatment options (12).

### NOS2/COX2/CD8 spatial localization defines the tumor immune landscape

Spatial localization analysis of NOS2/COX2/CD8 signatures (summarized in Supplementary Fig. S3C) was undertaken to further understand the phenotypic differences between 5-year surviving patients versus those who succumbed to disease. Notably, tumors from alive patients were fully inflamed with elevated CD8^+^ T-cell penetration deep into tumor cores. In contrast, the NOS2^−^/CD8^−^ phenotypes were observed in immune dessert regions, whereas the NOS2^+^ phenotypes in deceased patient tumors had stroma-restricted CD8^+^ T cells, and high tumor NOS2 expression was observed at the tumor margins. Pearson’s correlation analysis (*R*^2^ = 0.54, *P* = 0.0241) of these results suggests a possible progression from CD8^−^NOS2^−^ immune deserts to CD8^+^ NOS2^+^-restricted inflamed tumors (Supplementary Fig. S3D). These results show that tumors from surviving patients are highly inflamed with elevated CD8^+^ T cells that infiltrate deep into the tumor core. In contrast, deceased patient tumors are distinguished by two distinct phenotypes, cold immune desert regions, and inflamed tumors with stroma-restricted CD8^+^ T cells, where tumors expressing high NOS2 have migrated into the inflamed stroma, which determines outcome. Therefore, the spatial localization of NOS2 expressing tumor cells relative to CD8^+^ T cells could be key determinants of clinical outcomes (Fig. 2D).

**Figure 3:**
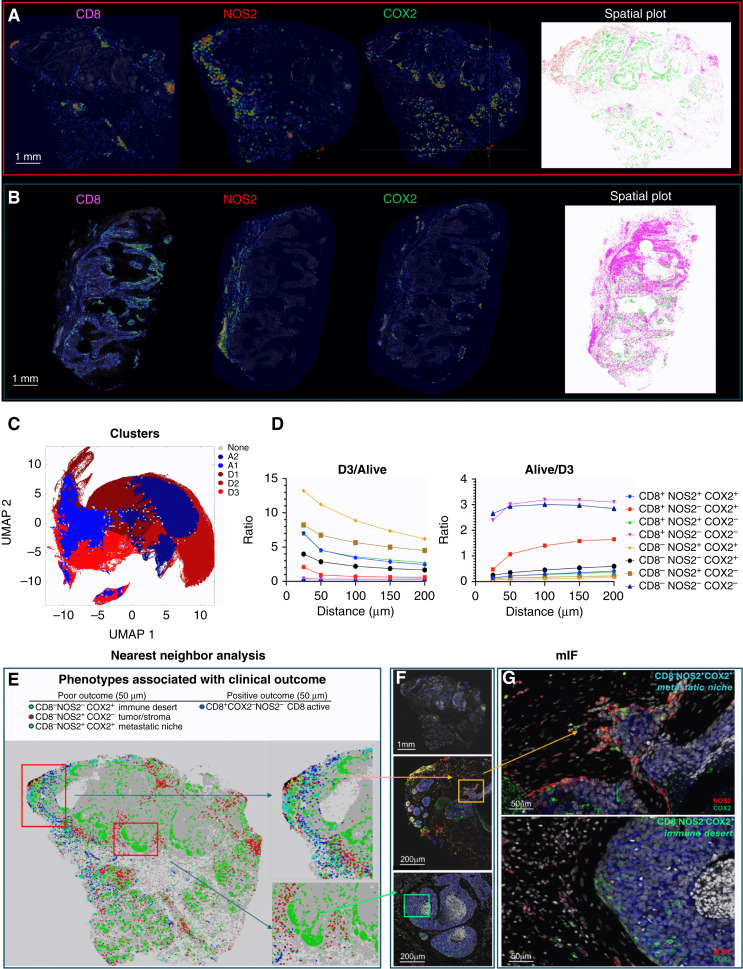
S-UMAP analysis for tumor NOS2/COX2 and CD8 expression. Density heat maps show spatially distinct tumor NOS2, COX2, and CD8 clustering in tumors from (**A**) deceased and (**B**) alive patients. Spatial distribution plots based upon positive pixels for CD8 (magenta), NOS2 (red; also shown in Fig. 1D), and COX2 (green; also shown in Fig. 1D) expression are shown. **C,** Masks show distinct neighborhood cluster prevalence in deceased (red) or alive (blue) patient tumors. **D,** Ratio of phenotype density profiles (i.e., neighborhood cluster density profiles) in the D3/alive as well as the alive/D3 demonstrates the predictive value of CD8^−^NOS2^+^COX2^+^ (yellow diamonds) and CD8^+^NOS2^−^COX2^−^ (purple triangle) phenotypes due to their vast differences in deceased vs. alive patient tumors. **E,** Spatial dot plot and nearest-neighbor analysis at 50 μm distances of the defined phenotypes with a predictive value. **F,** mIF of CD8 (magenta) NOS2 (red) and COX2 (green) expression in areas of interest (red square boxes) in the spatial dot plot shown in **E**. **F,** (Top) entire tumor image; **F**, (middle) a satellite or tumor budding metastatic niche (yellow box) expressing elevated tumor NOS2; (bottom) immune desert region. **G,** Enhanced magnification of these areas of interest (**F**, yellow and green boxes) shows a CD8^−^NOS2^+^COX2^+^ metastatic niche (top image; also shown in Fig. 1A) and CD8^−^NOS2^−^COX2^+^ immune desert (bottom image).

Previous studies have demonstrated the requirement of IFNγ and IL1/TNF cytokines for tumor NOS2/COX2 expression, in which correlations between tumor NOS2 and CD8 expressions were observed at the tumor stroma interface (3, 14, 21). To further explore this relationship relative to survival, spatial distribution and density heat maps were compared between tumors from deceased versus alive patients at 5-year survival, which demonstrated that tumor NOS2, tumor COX2 and CD8^+^ T-cell phenotypes occupy spatially distinct regions in the deceased patient tumors (Figs. 1D and 3A). A comparison of NOS2 density heat maps of the deceased patient tumors shows NOS2 expressing areas that are proximally orthogonal to COX2 and stroma-restricted CD8^+^ T cells. In contrast, tumors from surviving patients exhibited reduced NOS2 and COX2 clustering and increased CD8^+^ T-cell penetration into the tumor core (Figs. 1D and 3B). Thus, elevated tumor NOS2 and COX2 expression in tumors from deceased patients is spatially distinct and associated with limited CD8^+^ T-cell infiltration into the tumor core.

### Unsupervised spatial localization analysis

S-UMAP (19) was applied to all tumor images to validate spatial configurations independent of possible bias from visually supervised analysis. S-UMAP analysis of all cells across all patient tumors was used to identify and define unique cellular neighborhoods and nearest neighbors using the CD8^+/−^NOS2^+/−^COX2^+/−^ phenotypes summarized in Fig. 2 and Supplementary Fig. S3C. A neighborhood profile describing the density of cells of each phenotype as a function of distance from a given cell was calculated for every cell in all tumors (Supplementary Fig. S4A). The average neighborhood profiles of deceased and alive patient tumors (Supplementary Fig. S4A) revealed a greater than two-fold increase in the relative prevalence of CD8^−^NOS2^+^ phenotypes in cellular neighborhoods within tumors from deceased versus alive patients, which is in agreement with earlier results that correlate high NOS2 expression with worse outcome (4). However, this mean neighborhood analysis obscures the presence of distinct cellular niches that could have significant predictive power and provide potential therapeutic targets. Therefore, the S-UMAP of the complete set of cellular neighborhoods in all tumors (Supplementary Fig. S4B) was separated according to deceased versus alive patient tumors (Supplementary Fig. S4C and S4D), which revealed distinct cellular neighborhood distributions. The S-UMAP difference (Fig. 3C; Supplementary Fig. S4E and S4F), distinguishes the prevalent cellular neighborhoods in tumors from deceased (red) versus alive (blue) patients. K-means clustering (Supplementary Fig. S4G) of the cellular neighborhoods in deceased and alive tumor groups identified three cellular niches in deceased (D1, D2, and D3) and two in alive (A1 and A2) patient tumors that are summarized in Fig. 3C.

Next, the predictive value of these defined cellular niches was examined. Neighborhoods D1, D2, A1, and A2 are similar to the average cellular neighborhoods in tumors from deceased and alive patients (Supplementary Fig. S4A). Further comparisons between the five neighborhoods reveal that D1 and A1 are similar, as are D2 and A2. However, D3 is significantly different from A1 and A2 (Fig. 3C; Supplementary Fig. S4A and S4G). The average D3 neighborhood, which represents 23% of cells from deceased patient tumors (Supplementary Fig. S4H), has high densities of CD8^−^ cells (∼1,000 cells/mm^2^) as well as NOS2^+^ and COX2^+^ cells (Supplementary Fig. S4A). There is also a clear distance dependence (i.e., spatial clustering) of the phenotypes with maximum densities at nearest-neighbor distances of 25 μm (Fig. 3D). Therefore, the D3 neighborhood is locally defined (within 100 μm distances) by high densities of CD8^−^NOS2^−^COX2^+^ and CD8^−^NOS2^+^COX2^+^ cellular phenotypes, which correspond to immune desert regions and the metastatic niche as shown in Fig 3E–G and in Supplementary Fig. S4I. In contrast, A1 and A2 neighborhoods derived from alive patient tumors (Supplementary Fig. S4H) had relatively high densities (up to >1,000 cells/mm^2^ in A1) of the CD8^+^NOS2^−^COX2^−^ phenotype (Supplementary Fig. S4I), which corresponds with fully inflamed infiltrating CD8^+^ T cells that penetrated deep into the tumor core of low NOS2/COX2-expressing tumors (Supplementary Fig. S4J). These findings corroborate the visually observed phenotypes described above and support the idea that CD8^+^ T-cell status impacts outcome, which is influenced by the spatial landscape of tumor NOS2/COX2 expression. Importantly, the unbiased S-UMAP approach comes to the same conclusions as the observer-driven approach and both are thus supportive of one another.

The spatial landscape and ratio of phenotype prevalence between the D3 and alive neighborhoods (Fig. 3D; Supplementary Fig. S4A and S4B) visualize the vast difference between neighborhood compositions in the metastatic niche (CD8^−^NOS2^+^COX2^+^) versus fully inflamed tumors (CD8^+^NOS2^−^COX2^−^) with high CD8^+^ T-cell density. Fig. 3D shows the ratio of phenotype profiles in D3 neighborhoods relative to all alive neighborhoods (A1 + A2). This analysis reveals ∼10-fold increase in local densities of CD8^−^NOS2^+^COX2^+^ and CD8^−^NOS2^+^COX2^−^ cells (metastatic niche), a >5-fold increase in CD8^+^NOS2^+^ cells (restricted inflamed tumor), and a >2-fold increase in CD8^−^NOS2^−^COX2^+^ cells (immune desert). D3 neighborhoods have less than 50% of the prosurvival phenotype CD8^+^NOS2^−^COX2^−^ than A1 and A2 neighborhoods.

In addition to the metastatic niche that predicted poor clinical outcomes (Fig. 3E), stroma-restricted CD8^+^NOS2^+^COX2^+^ and CD8^+^NOS2^+^COX2^−^ phenotypes are also elevated by approximately 5-fold in D3 relative to A1 and A2 neighborhoods. Their ratios also decrease with distance more than other phenotypes, supporting the stroma restriction interpretation. Although CD8^+^NOS2^+^COX2^+^ and CD8^+^NOS2^+^COX2^−^ phenotypes comprise a lower density of cells in cluster D3, their presence could have a predictive value. Therefore, the CD8^+^ status of these phenotypes in cluster D3 could indicate an increased presence of metastatic or stem cell niches near stroma-restricted CD8^+^ T cells, in agreement with the work of Stein and colleagues (29), who showed that proximal CD8^+^ T cells can induce cancer stemness in the murine model. The lack of CD8^+^ T-cell penetration from the tumor/stroma interface into the tumor core supports observations of increased stroma-restricted CD8^+^ T cells as well as immune desert regions lacking CD8^+^ T cells in deceased patient tumors (Figs. 1A and 3E–G; Supplementary Fig. S4J, respectively). This more focused approach defines a spatial relationship between tumor NOS2 and COX2 expression and CD8^+^ T cells where restricted CD8^+^ T cells that are excluded from the tumor are associated with elevated tumor NOS2 expression, whereas abated CD8^+^ T-cell penetration into the tumor core corresponds with tumor COX2-expressing immune desert regions (Fig. 3E–3G; Supplementary Fig. S5A–S5C). Taken together, these analyses show that tumor NOS2/COX2 and CD8^+^ T-cell spatial orientation defines distinct cellular neighborhoods with predictive power.

**Figure 4:**
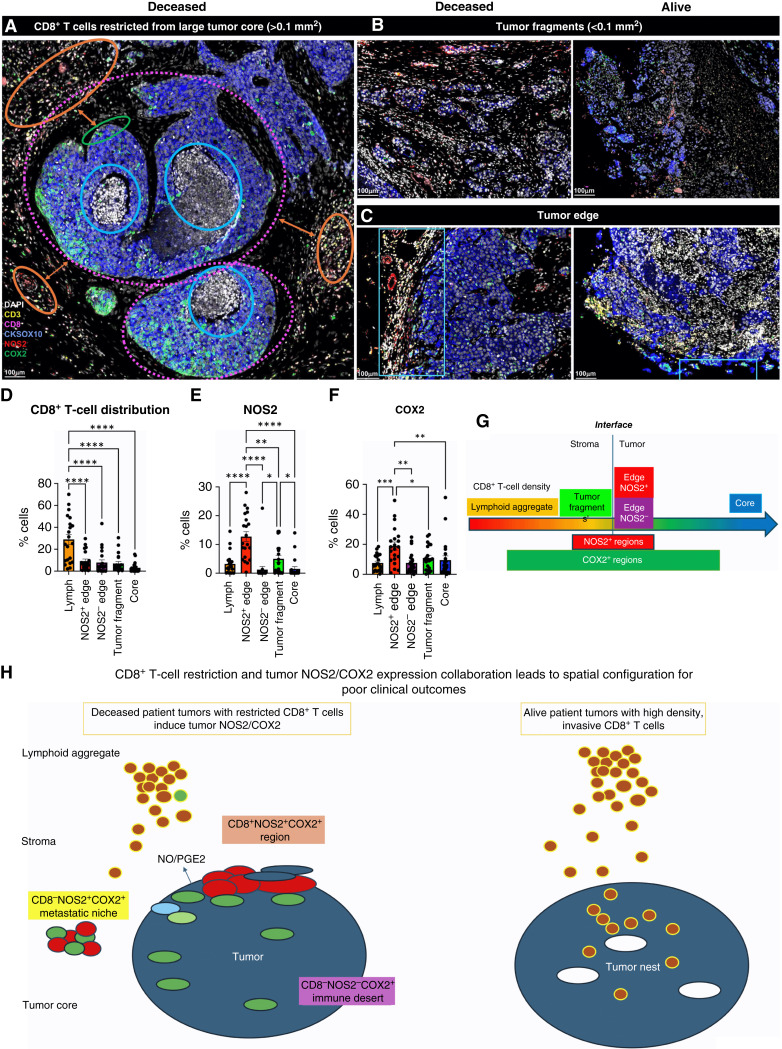
Defined CD8 NOS2 and COX2 spatial landscape. Five basic regions showing (**A**) margin or stroma-restricted lymphoid aggregates (orange circles) in which a gap of 50 μm (double orange arrow) between CD3^+^ T-cell aggregates and the CKSOX10^+^ tumor edge (green circle) was observed. Blue and dashed magenta circles identify immune desert regions lacking CD8^+^ T cells. **B,** Tumor fragmentation or satellite region. **C,** Tumor edge with proximal stroma regions. **D,** Significant differences in % cell composition is shown for CD8^+^ T cells as well as (**E**) NOS2^+^ and (**F**) COX2^+^ tumor cells. **G,** Graphic summary of tumor NOS2/COX2 landscape and CD8^+^ T-cell regional distributions with respect to the tumor–stroma interface. **H,** Spatial architecture of predictive phenotypes in tumors from deceased vs. alive patients. *, P ≤ 0.05; **, *P* ≤ 0.01; ***, *P* ≤ 0.001; ****, *P* ≤ 0.0001.

Next, the spatial significance of CD8^+^ T cells relative to tumor NOS2/COX2 was examined with respect to known pathologic features of the TME and is summarized in Supplementary Table S1. Figure 4A–C show five distinct annotated areas including (i) lymphoid aggregates (Fig. 4A orange circles); (ii) regions associated with larger tumor nests (>0.05 mm^2^; Fig. 4A magenta dashed circle); (iii) the tumor core (Fig. 4A blue circle); (iv) areas of tumor fragmentation (<0.1 mm^2^; Fig. 4B); and (v) NOS2^+^ (Fig. 4C blue box) and NOS2^−^ tumor edges, defined as the region at the tumor interface of larger tumor nests. The lymphoid aggregates were conglomerates of CD3^+^ lymphoid cells at the tumor margin in tumors from both deceased and surviving patients. The CD8^+^ T-cell distributions in these defined regions were examined in all tumors, where distribution analysis showed elevated CD8^+^ T-cell populations in lymphoid aggregates, whereas the lowest CD8^+^ T-cell distribution was observed in the tumor core of immune desert regions (Fig. 4D). Further stratification for survival revealed that the CD8^+^ T-cell distribution in alive patient tumors was approximately 4-fold higher in the tumor core when compared with deceased patient tumors (Supplementary Table S2). Immune deserts were previously defined as 100 cells/mm^2^, which is approximately 1% cells (13). This again confirms that CD8^+^ T cells in tumors from deceased patients are restricted and marginalized. In contrast, increased CD8^+^ T-cell infiltration was observed in tumors from alive patients at 5-year survival. Thus, the result that CD8^+^ T cells are stroma-restricted in deceased patient tumors but highly infiltrate tumor cores in alive patient tumors is consistent with the nearest-neighbor analysis in the S-UMAP shown in Fig. 3 and Supplementary Fig. S4J.

The spatial localization of tumor NOS2/COX2 expression versus CD8^+^ T cells shows a different pattern within the defined regions. Tumor NOS2 density was evaluated at the tumor edge (Fig. 4E) where tumor satellite regions indicative of invasive tumor cells (14) contained significantly higher NOS2 expression, whereas NOS2 was lowest in lymphoid aggregates and the tumor core (Fig. 4E). Importantly, the tumor core is nearly devoid of NOS2 (Fig. 4E) as well as CD8^+^ T cells, supporting the idea that NOS2 resides at the tumor/stroma interface as previously described (14). NOS2 was significantly higher in the tumor satellite regions when compared with the tumor core (Fig. 4E). In contrast, COX2 expression was more evenly distributed in these defined regions (Fig. 4F). COX2 was significantly higher in association with the NOS2^+^ tumor edge but was also found in the tumor core and stroma-restricted lymphoid aggregates (Fig. 4F). The analysis shows clear distinction and regional distribution of CD8^+^ T cells and tumor NOS2 and COX2 as summarized in Fig. 4G that correlates with survival (Fig. 1).

Tumors from deceased patients exhibit gaps, separating larger tumor nests from stroma-restricted CD8^+^ T cells, which was not observed in tumors from surviving patients. As shown above, both NOS2 and COX2 can be expressed on larger tumors, and NOS2^+^/COX2^+^ foci are associated with areas of inflammation proximal to stroma-restricted CD8^+^ T cells as previously described (14). Larger tumor nests (>0.05 μm^2^) exhibit gaps (average of 50 μm) between tumor and lymphoid cells (Fig 4A orange arrows). Stroma-restricted lymphoid aggregates average 500 to 1,000 μm from NOS2^+^ and/or COX2^+^ tumor edges. Stroma-restricted CD8^+^ T cells were observed 50 to 100 μm from the tumor margin of NOS2^−^COX2^+^ tumor edges. Although COX2 is expressed at the edge of immune deserts, its expression is abated deeper at the core of immune deserts. These observations could indicate that COX2/PGE2 may serve as a barrier preventing CD8^+^ T-cell infiltration into the tumor core (Supplementary Fig. S3B 4T1 tumors), thus facilitating the development of an immune desert, which is consistent with increased CD8^+^ T-cell penetration into the core associated with COX inhibition by NSAID treatment (11). These observations further support a role of tumor NOS2 and COX2 expression during progression from immune desert to inflamed foci regions.

The above results show two principle CD8^+^ T-cell spatial orientations associated with tumor NOS2/COX2 expression, which are inflamed, stroma-restricted lymphoid, and immune deserts devoid of lymphoid cells (Fig. 4H). The immune desert is COX2^+^ but NOS2^−^; however, NOS2^+^ and COX2^+^ tumor satellites form in the inflamed areas near stroma-restricted CD8^+^ T cells that produce IFNγ (14). Importantly, these NOS2^+^ tumor satellites exhibit increased elongation and migration consistent with increased tumor metastatic potential (14). Elevated tumor NOS2 and COX2 promote a feed forward mechanism that drives cancer cell phenotypes with metastatic, chemoresistant, and CSC properties (3, 30). Tumor CD44v6 and EpCAM expressions have been used as clinical CSC markers in breast and other cancers, correlating with metastasis, circulating tumor cells, CSC, and chemoresistance (31). Next, these markers were used to spatially identify CSC niches in the TME during disease progression.

**Figure 5:**
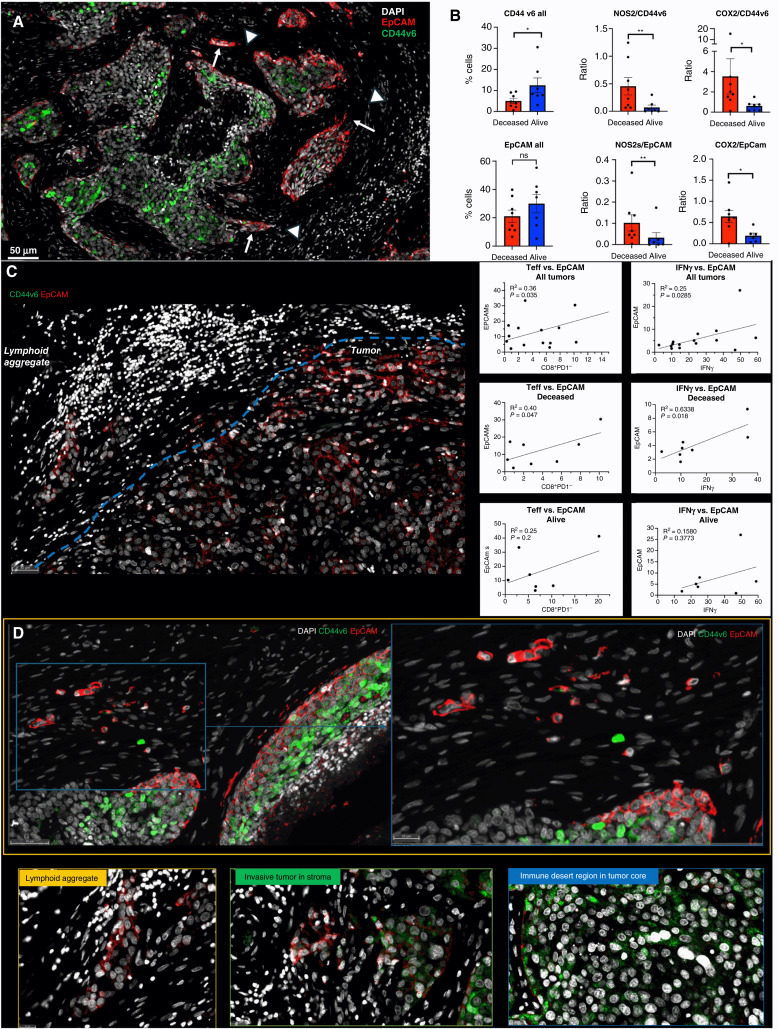
Spatial landscape of CD44v6 and EpCAM expression relative to tumor NOS2/COX2 expression and survival. **A,** Enriched regions showing spatially distinct CD44v6 and EpCAM expression. **B,** Quantification of CD44v6 and EpCAM as well as ratios of NOS2 or COX2 to CD44v6 or EpCAM, respectively, in tumors from deceased vs. alive patients. Significance was determined using the Mann–Whitney test, in which *, *P* < 0.05; **, *P* = 0.007. **C,** Lymphoid aggregate and EpCAM^+^ tumor cell boarder. Pearson’s correlation coefficient shows significant associations between T effector cells or IFNγ and EpCAM expression in tumors from deceased vs. alive patients. **D,** Spatial landscape of CD44v6 and EpCAM expression in annotated tumor, lymphoid aggregate, invasive tumor/stroma, and immune desert regions. These regions show EpCAM^+^ and CD44v6^+^ tumor cells that have invaded the stroma.

**Figure 6:**
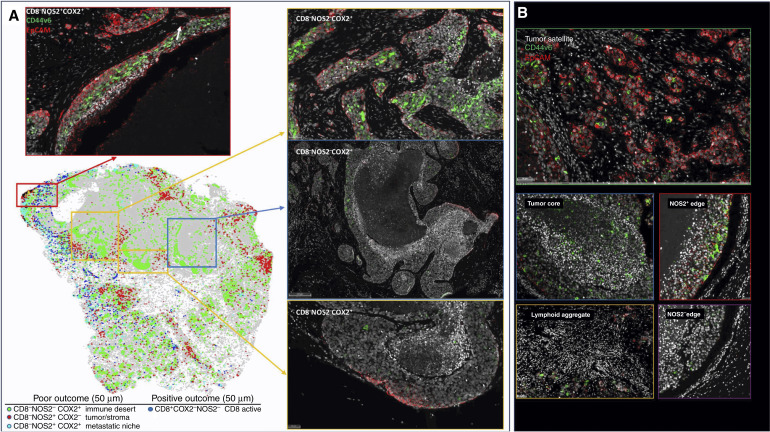
S-UMAP and regional annotations identify cellular neighborhoods of interest with respect to the tumor CSC markers EpCAM and CD44v6. **A,** Unsupervised analysis of S-UMAP highlighting distinct EpCAM and CD44v6 expression in specific magnified regions (also shown in Fig. 5D); red box showing CD8^−^NOS2^+^COX2^+^ phenotype in the metastatic niche (cyan) in a stroma-restricted inflamed region; yellow and blue boxes show CD8^−^NOS2^−^COX2^+^ phenotypes in immune desert regions. **B,** Supervised analysis showing distinct EpCAM and CD4v6 expression in regions containing tumor satellites, tumor core, NOS2^+^ edge, lymphoid aggregates, and NOS2^−^ edge.

Spatial localization of tumor CD44v6 and EpCAM expression showed distinct expression patterns (Fig. 5A). Univariant analysis demonstrated significantly higher CD44v6 expression in tumors from alive patients, whereas EpCAM did not change significantly with respect to survival (Fig. 5B CD44v6 all, EpCAM all). Given that CD44v6 is expressed on lymphocytes and modulates their functional activity (32–35) and that tumor NOS2 and COX2 expression modulates CD8^+^ T-cell penetration into the tumor core (11), potential roles for tumor NOS2 and COX2 relative to CD44v6 and EpCAM expression were evaluated by comparing ratios of tumor NOS2 and COX2 to CD44v6 and EpCAM. The ratios of NOS2/CD44v6, NOS2/EpCAM, COX2/CD44v6, and COX2/EpCAM were significantly elevated in tumors from deceased patients when compared with those of alive patients at 5-year survival, as shown in Fig. 5B. These results suggest that elevated tumor NOS2 and COX2 could influence the effects of EpCAM and CD44v6 relative to clinical outcomes. This is supported by cell culture experiments showing NO-induced CD44v6 in MB231 cells and cytokine-induced EpCAM expression in MCF-7 and MB468 cells (Supplementary Fig. S6A and S6B). We recently demonstrated significant correlations between tumor NOS2 and COX2 expression and both stroma-restricted CD8^+^ T effector cells and secreted IFNγ in these tumors (14). Next, we used Pearson’s correlation coefficients to explore potential associations between stroma-restricted CD8^+^ T effector cells and IFNγ identified in lymphoid aggregates with the cancer stemness biomarker EpCAM and CD44v6 expression levels; significant *R*^2^ values were determined for CD8^+^ T effector cells or IFNγ versus EpCAM in tumors from deceased patients (Fig. 5C). In contrast, significant correlations were not observed for CD44v6 as this biomarker was largely identified is immune desert regions devoid of CD8^+^ T cells (Fig. 5D). The significant correlations identified between CD8^+^ T effector cells, IFNγ, and EpCAM suggest that EpCAM could be induced by INFγ in stroma-restricted lymphoid aggregate regions. To explore this possibility, the spatial geography of EpCAM as well as CD44v6 was examined using information from the S-UMAP analysis shown in Fig. 3E, which identified regional CD8^−^NOS2^+^COX2^+^, CD8^−^NOS2^+^COX2^−^, and CD8^−^NOS2^−^COX2^+^ cellular phenotypes in deceased patient tumors. When compared with Fig. 3E–G, Figs. 5D and 6A show EpCAM expression on the tumor edge in NOS2^+^COX2^+^ niches. In contrast, CD44v6 is predominantly localized in COX2^+^ immune desert regions of the tumor core. In support of this observation, CD44v6 is significantly reduced in INDO-treated 4T1 tumor-bearing mice (Supplementary Fig. S6C). The S-UMAP analysis revealed highly significant correlations between EpCAM in lymphoid restricted regions, where elevated IFNγ and NOS2 were expressed, either at the tumor edge or in tumor satellites areas (Fig. 6B). Although EpCAM is expressed with sporadic CD44v6 expression in lymphoid aggregate areas, EpCAM expression is reduced along tumor NOS2^−^ edges. These results suggest a progression from immune desert regions expressing COX2 and CD44v6 to inflammatory NOS2^+^ regions, where EpCAM expression is predominant.

**Figure 7:**
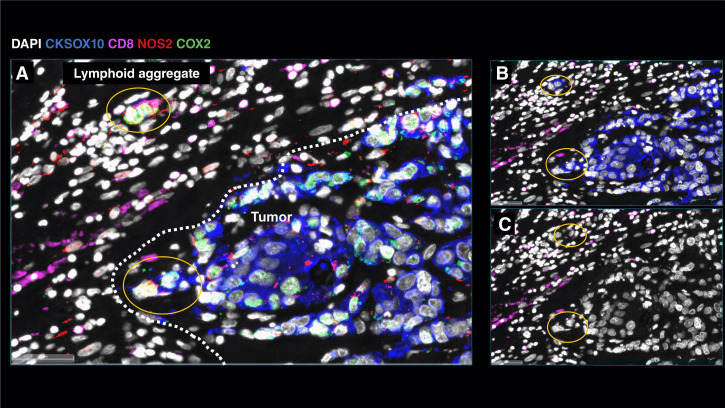
Metastatic niche showing invasive tumor edge proximal to lymphoid aggregates. **A,** Composition of tumor marker CKSOX10 (blue), NOS2 (red), COX2 (green), and CD8 (magenta), in which NOS2^+^ and COX2^+^ cells (yellow circles) have migrated away from larger tumor lesion and invaded into the stroma. **B,** CKSOX10 tumor marker alone relative to CD8^+^ T-cell aggregate. **C,** DAPI with CD8 expression.

Further examination of tumor cells localized in stroma regions provided structural details implicating progression to metastatic disease, where small clusters of elongated EpCAM positive cells that could represent metastatic phenotypes were identified along NOS2^+^ edges (Figs. 5A and 6A white arrows; ref. 14). As seen above, rich EpCAM areas show small clusters of elongated EpCAM^+^ cells in the stroma near lymphoid aggregates (Fig. 5A white arrowhead) indicating tumor cell migration in the vicinity of restricted lymphoid patches that induce tumor NOS2 and COX2 expression (14). In contrast to elongated EpCAM^+^ phenotypes observed near restricted lymphoid aggregates, Fig. 5D, shows CD44v6 that is expressed predominantly in the immune desert epithelial tumor core. These results suggest that tumor NOS2, COX2, and CD8 expressions demonstrate elongated tumor NOS2 and COX2 clusters that are spatially localized in stroma-restricted lymphoid aggregates (Fig. 7A) near the NOS2^+^ tumor edge (Fig. 7A). The coexpression of NOS2^+^ and CKSOX10^+^ tumor cells, as well as CD8^+^ lymphoid aggregate, is discerned in Fig. 7A–C, respectively. Taken together, tumor NOS2 and COX2 expression collaborates during the metastatic process, which may involve at least in part the upregulation of EpCAM expression (36, 37).

## Discussion

The strong association between tumor NOS2 and COX2 coexpression with survival in ER^−^ breast cancer suggests that these enzymes and their products are key drivers of poor clinical outcomes (2). Previous work has shown that NOS2-derived NO and COX2-derived PGE2 collaborate in a feedforward manner to activate multiple oncogenic pathways that promote metastasis, cancer stemness, and immunosuppression, which are all markers of disease progression (2, 38). These earlier observations are extended herein by the identification of unique EpCAM^+^ and CD44v6^+^ cellular neighborhoods that border NOS2 and COX2 high regions. Given that EpCAM and CD44v6 are markers of metastasis and cancer stemness, these observations support the role(s) of tumor NOS2 and COX2 expression as key targets of disease progression in breast and other tumors (37, 39). Moreover, the orthogonal expression of tumor NOS2 and COX2 in distinct cells and regions within the TME suggests that the spatial configuration of these enzymes has important roles during intercellular communication and disease progression. Herein we show spatial associations between regional clustering of tumor NOS2 and specific EpCAM^+^ cellular neighborhoods along the tumor margin and in the stroma in tumors from deceased patients at 5-year survival. Elevated tumor NOS2 expression and the intracellular NO levels that activate major oncogenic pathways through S-nitrosation and nonheme iron is consistent with 300 μmol/L levels determined *in vitro* (2, 3, 30). The levels of NO critical for activation of specific pathways are dependent on three factors including the rates of NO production and consumption, as well as the frequency or clustering of NOS2-expressing cells. Previous studies have shown that the higher frequency or number of clustered NOS2 expressing cells is directly proportional to the local NO concentration (11, 21). These regions of elevated tumor NOS2 expression are spatially consistent with observed metastatic niches defined by elevated EpCAM expression.

The regulation of tumor NOS2 in human cancer cells has been somewhat of a mystery. In murine systems, the requirement of IFNγ for tumor Nos2 expression has been shown, which was amplified by other cytokines including IL1β and TNFα (21). In comparison, human NOS2 expression was detected under the same conditions *in vitro* but was considerably lower (3%–5%) when compared with Nos2 expression (40%–100%) in murine 4T1 tumor cells (14, 21). Regional NOS2 expression levels *in vivo* are far higher (∼30%–40%). CD8^+^ T cells provide a source of IFNγ in the TME, and their increased presence is generally predictive of improved clinical outcomes (40). Herein, elevated CD8^+^ T cell/tumor ratio also correlated with an improved clinical outcome. Moreover, the results herein show an important spatial correlation, in which the stroma restriction of CD8^+^ T and increased tumor NOS2 and COX2 expression correlated with poor survival. These inflamed regions of stroma-restricted CD8^+^ T cells create transient areas of locally increased NO/PGE2, which promote increased oncogenic signaling (2). Although tumor-infiltrating CD8^+^ T cells augment therapeutic efficacies, their spatial restriction in tumor stroma is predictive of poor survival, in which increased IFNγ and cytokine stimulated tumor NOS2 and COX2 expression creates a cellular configuration culminating in abated CD8^+^ T-cell infiltration as well as the development of metastatic and CSC niches (13, 14). In contrast, tumors from surviving patients at 5-year postdiagnosis exhibited low, sporadic tumor NOS2/COX2 expression and elevated CD8^+^ T-cell infiltration into the tumor, which promotes tumor eradication by perforins and granzyme B in a cell-to-cell contact manner (11). Therefore, the relationship between elevated tumor NOS2/COX2 expression and abated CD8^+^ T-cell tumor infiltration implicates the importance of the spatial biology of tumor NOS2 and COX2 expression. Importantly, CD8^+^ T-cell restriction provides a therapeutic barrier that induces regional tumor NOS2 and COX2 expression, metastasis, and cancer stemness.

Examination of different cellular niches demonstrated unexpected regional differences in the expression of CSC markers EpCAM and CD44v6, in which EpCAM^+^ cells were spatially aligned with NOS2 expressing tumor cells near stroma-restricted lymphoid aggregates at the tumor margin or in the tumor stroma. In contrast, CD44v6 was expressed in immune desert regions in the tumor core surrounded by COX2. EpCAM and CD44v6 can be induced by several factors including cytokines. Although EpCAM can be induced by IL8 and IFNγ, it is inhibited by TNFα and IL6 (41–43). In contrast, IL6 and TNFα can induce CD44v6, which could in part explain the distinct EpCAM and CD44v6 spatial localization (43). Previously, we showed that IL8 was induced in cancer cells by IFNγ and NOS2 (4, 14). In contrast, CD44v6 in some cancers is inhibited by IFNγ but induced by TNFα, IL1α, IL1β, and IL6 (44). It was also shown that IL1 and TNFα induced by NO can, in separate cells, induce COX2 (2, 14). Also, COX2-derived PGE2 induces IL6 (2). Taken together, the differential response to cytokines associated with tumor NOS2/COX2 expression can in part explain the spatially distinct expression of CSC markers: EpCAM was increased along inflamed NOS2^+^ edges, whereas COX2^+^ regions surrounded CD44v6 expressing cells in immune desert regions.

In summary, this work describes the influence of the tumor NOS2/COX2 landscape on antitumor immunity and the development of cancer metastatic and stem cell niches. We have reported oncogenic signaling pathways induced by NO and PGE2 in breast cancer cells grown in culture. However, these experiments lacked the spatial organization of the tumor immune microenvironment *in situ* and how it can be influenced by regional tumor NOS2 and COX2 expression. Enhanced *T*_*eff*_ cell infiltration into the tumor epithelium is required for effective antitumor immunity and improved therapeutic efficacy. We show here that COX2 expression at the tumor margin limits CD8^+^ T-cell infiltration into the tumor core, which involves at least in part reduced cytokine and chemokine expression (IRF8, CLEC9a, CXCL9, CXCL10, CXCL11, and IL27) that promotes directional immune cell migration. The resulting stroma-restricted CD8^+^ T cells are associated with an immunosuppressive microenvironment. In addition, stroma-restricted CD8/CD4 T cells supply IFNγ and TNF/IL1 to the tumor nest (14), which further promotes tumor NOS2/COX2 expression at the tumor margin that leads to the formation of metastatic and CSC niches that are regionally distinct. In addition, these results demonstrate spatially distinct tumor NOS2, COX2, and CSC biomarker expression, suggesting that the production of different diffusible cytokines can shape the cellular neighborhoods that drive disease progression. The above findings show that the NOS2/COX2 spatial configuration proximal to CD8^+^ T cells creates cellular niches that promote metastasis and cancer stemness. The clustering of inflamed NOS2^+^/EpCAM^+^ cellular neighborhoods promotes increased regional NO flux that drives metastatic phenotypes and poor clinical outcomes. In contrast, COX2^+^/CD44v6^+^ cellular neighborhoods localized in immune desert regions of the tumor core promote immune suppression and chemoresistant tumor phenotypes that could be exploited by NSAIDs or chimeric antigen receptor T-cell therapy targeting CD44v6 (45, 46). Importantly, the characterization of these phenotypes not only have strong predictive power but can be used to design novel therapies that include clinically available NSAIDs and NOS inhibitors, which could improve clinical outcomes.

## Supplementary Material

Supplementary Table I Supplementary Table I summarizes pathological features of tumor immune microenvironment where NOS2+ inflamed regions are significantly higher in tumors from Deceased patients.

Supplementary Table II Supplementary Table II summarizes %CD8+ T cells in designated regions of the tumors.

Supplementary Figure 1 Tumor NOS2 and COX2 expression induced by IFNg and TNFa

Supplementary Figure 2 Tumor analysis of NOS2 and COX2.

Supplementary Figure 3 Immune and tumor quantification

Supplementary Figure 4 Supplementary Fig. 4. Supplementary Fig. 4. Spatial UMAP analysis of CD8+/-NOS2+/-COX2+/- phenotypes in Deceased vs Alive patient tumors. Single cell neighborhood profile summary.

Supplementary Figure 5 Spatial dot plot of CD8+/-NOS2+/-COX2+/- phenotypes in tumor from deceased patient.

Supplementary Figure 6 Modulation of EpCAM and CD44v6+.
