# Supplementary material for: Tumor NOS2 and COX2 Spatial Juxtaposition with CD8+ T Cells Promote Metastatic and Cancer Stem Cell Niches that Lead to Poor Outcome in ER− Breast Cancer
Source: Cancer Res Commun. 2024 Oct 23;4(10):2766–82. doi: 10.1158/2767-9764.CRC-24-0235 (PMC11497117; doi:10.1158/2767-9764.CRC-24-0235)
Supplement: Supplementary Table I — summarizes pathological features of tumor immune microenvironment where NOS2+ inflamed regions are significantly higher in tumors from Deceased patients. [file crc-24-0235_supplementary_table_i_suppst1.docx]

**Supplementary Table I**

Pathological Features of Tumor Immune Microenvironment in

Tumors from Deceased vs Alive Patients

Region %Deceased (n = 11) %Alive (n = 10) p value

1. Lymphoid Aggregate 12 9 ns
2. Large Tumor Nests 31 26 ns
3. Tumor Fragmentation 9 10 ns
4. Tumor Core 21 15 ns
5. NOS2^-^ Tumor Edges 7 7 ns

NOS2^+^ Tumor Edges 8 4 0.054

*NOS2^+^ Inflamed 36 0 0.002*

**Supplementary Table I** summarizes pathological features of tumor immune microenvironment where NOS2^+^ inflamed regions are significantly higher in tumors from Deceased patients.
