# Supplementary material for: Tumor NOS2 and COX2 Spatial Juxtaposition with CD8+ T Cells Promote Metastatic and Cancer Stem Cell Niches that Lead to Poor Outcome in ER− Breast Cancer
Source: Cancer Res Commun. 2024 Oct 23;4(10):2766–82. doi: 10.1158/2767-9764.CRC-24-0235 (PMC11497117; doi:10.1158/2767-9764.CRC-24-0235)
Supplement: Supplementary Table II — summarizes %CD8+ T cells in designated regions of the tumors. [file crc-24-0235_supplementary_table_ii_suppst2.docx]

**Supplementary Table II**

%CD3+CD8^+^ T Cells in Designated Regions

Region Deceased Alive

Lymph 25.0 37.0

Tumor Fragments 5.0 10.0

NOS2+ 8.8 12.0

NOS2- 7.0 8.0

Core 1.0 4.4

**Supplementary Table II** summarizes %CD8^+^ T cells in designated regions of the tumors.
