# Supplementary material for: Tumor NOS2 and COX2 Spatial Juxtaposition with CD8+ T Cells Promote Metastatic and Cancer Stem Cell Niches that Lead to Poor Outcome in ER− Breast Cancer
Source: Cancer Res Commun. 2024 Oct 23;4(10):2766–82. doi: 10.1158/2767-9764.CRC-24-0235 (PMC11497117; doi:10.1158/2767-9764.CRC-24-0235)
Supplement: Supplementary Figure 1 — Tumor NOS2 and COX2 expression induced by IFNg and TNFa [file crc-24-0235_supplementary_figure_1_suppsf1.pptx]

## Slide 1
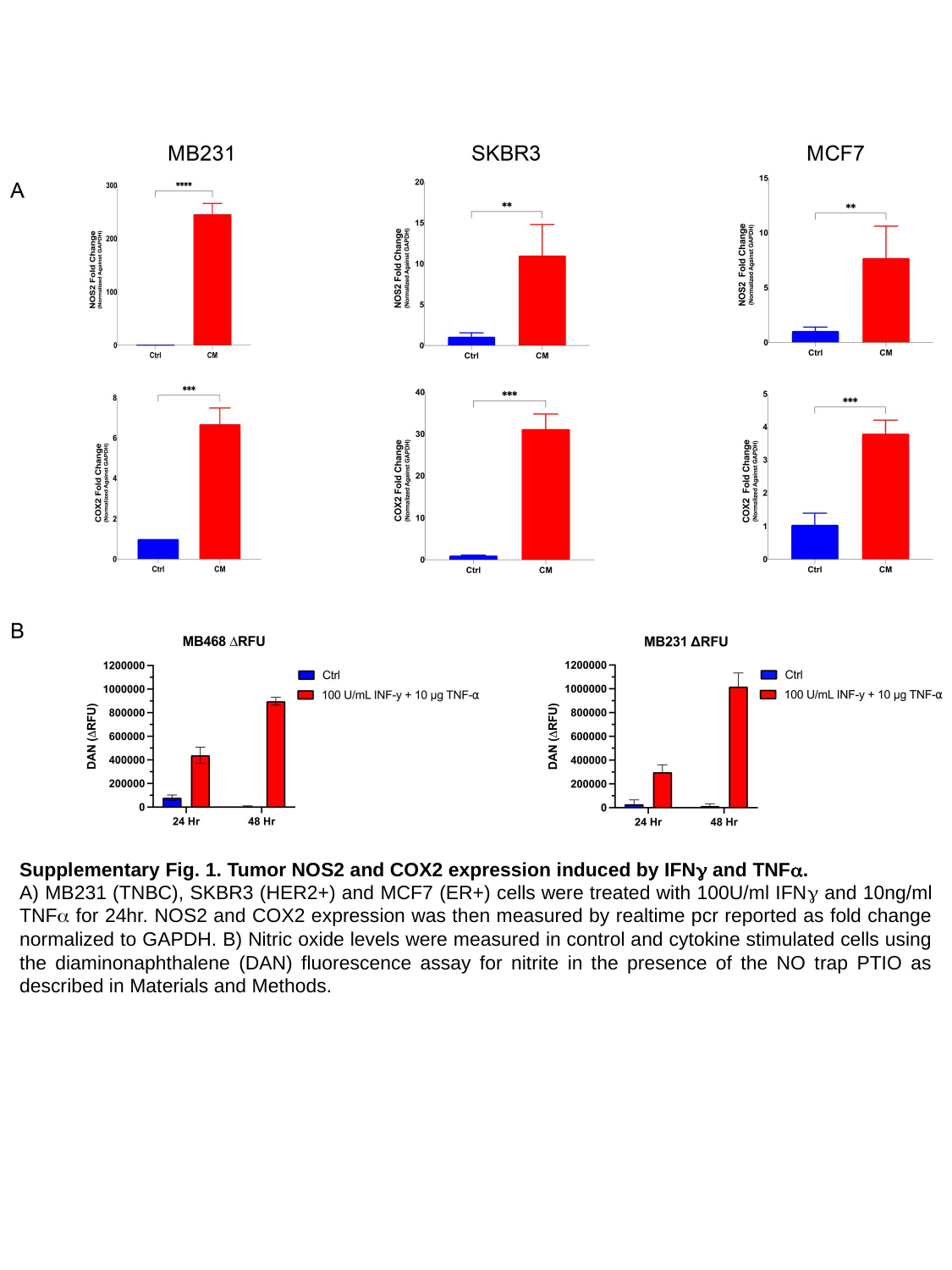

Supplementary Fig. 1. Tumor NOS2 and COX2 expression induced by IFNg and TNFa.
A) MB231 (TNBC), SKBR3 (HER2+) and MCF7 (ER+) cells were treated with 100U/ml IFNg and 10ng/ml TNFa for 24hr. NOS2 and COX2 expression was then measured by realtime pcr reported as fold change normalized to GAPDH. B) Nitric oxide levels were measured in control and cytokine stimulated cells using the diaminonaphthalene (DAN) fluorescence assay for nitrite in the presence of the NO trap PTIO as described in Materials and Methods.
