# Supplementary material for: Tumor NOS2 and COX2 Spatial Juxtaposition with CD8+ T Cells Promote Metastatic and Cancer Stem Cell Niches that Lead to Poor Outcome in ER− Breast Cancer
Source: Cancer Res Commun. 2024 Oct 23;4(10):2766–82. doi: 10.1158/2767-9764.CRC-24-0235 (PMC11497117; doi:10.1158/2767-9764.CRC-24-0235)
Supplement: Supplementary Figure 2 — Tumor analysis of NOS2 and COX2. [file crc-24-0235_supplementary_figure_2_suppsf2.pptx]

## Slide 1
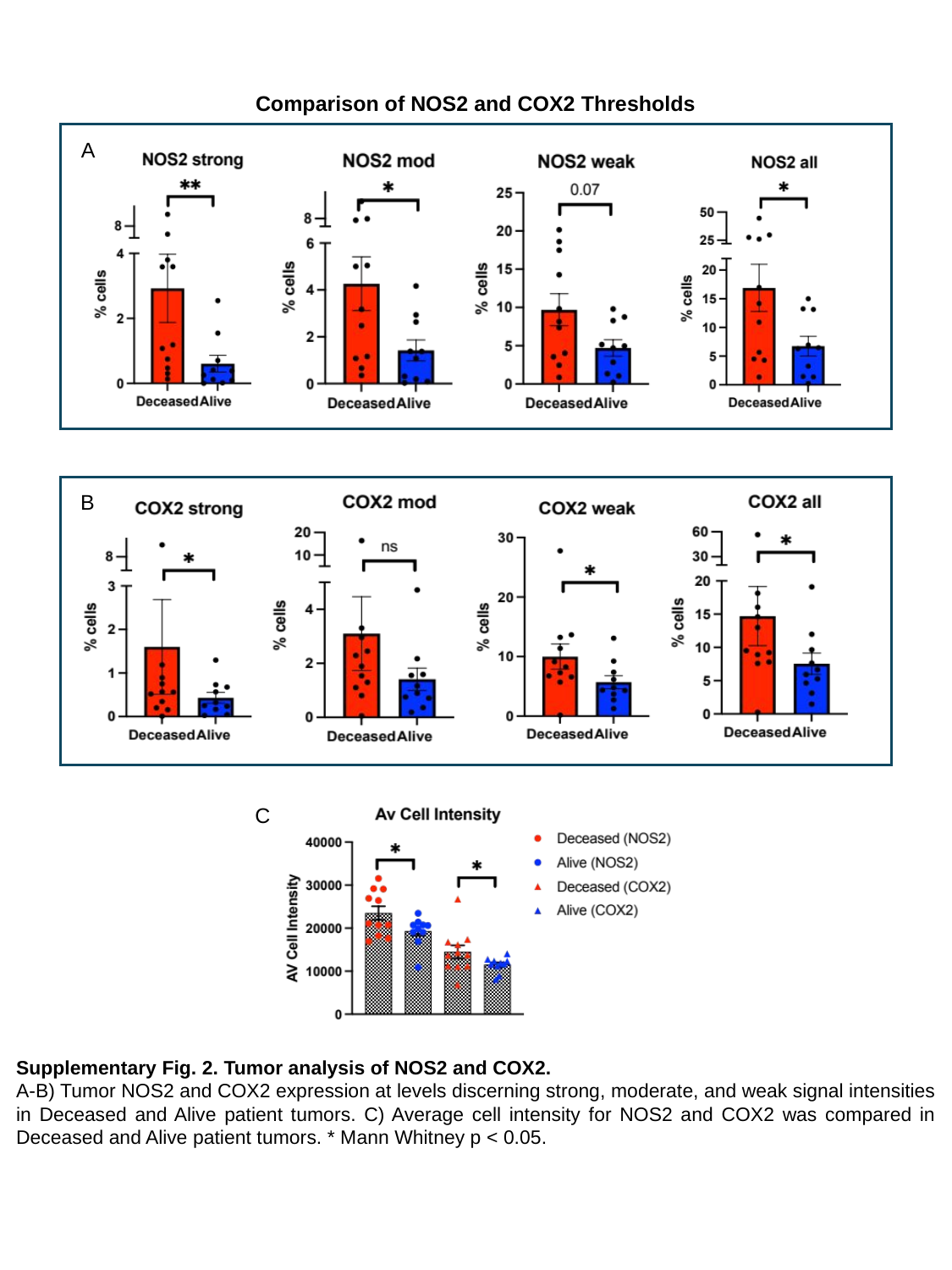

Comparison of NOS2 and COX2 Thresholds
A
B
C
Supplementary Fig. 2. Tumor analysis of NOS2 and COX2.
A-B) Tumor NOS2 and COX2 expression at levels discerning strong, moderate, and weak signal intensities in Deceased and Alive patient tumors. C) Average cell intensity for NOS2 and COX2 was compared in Deceased and Alive patient tumors. * Mann Whitney p < 0.05.
