# Supplementary material for: Tumor NOS2 and COX2 Spatial Juxtaposition with CD8+ T Cells Promote Metastatic and Cancer Stem Cell Niches that Lead to Poor Outcome in ER− Breast Cancer
Source: Cancer Res Commun. 2024 Oct 23;4(10):2766–82. doi: 10.1158/2767-9764.CRC-24-0235 (PMC11497117; doi:10.1158/2767-9764.CRC-24-0235)
Supplement: Supplementary Figure 3 — Immune and tumor quantification [file crc-24-0235_supplementary_figure_3_suppsf3.pptx]

## Slide 1
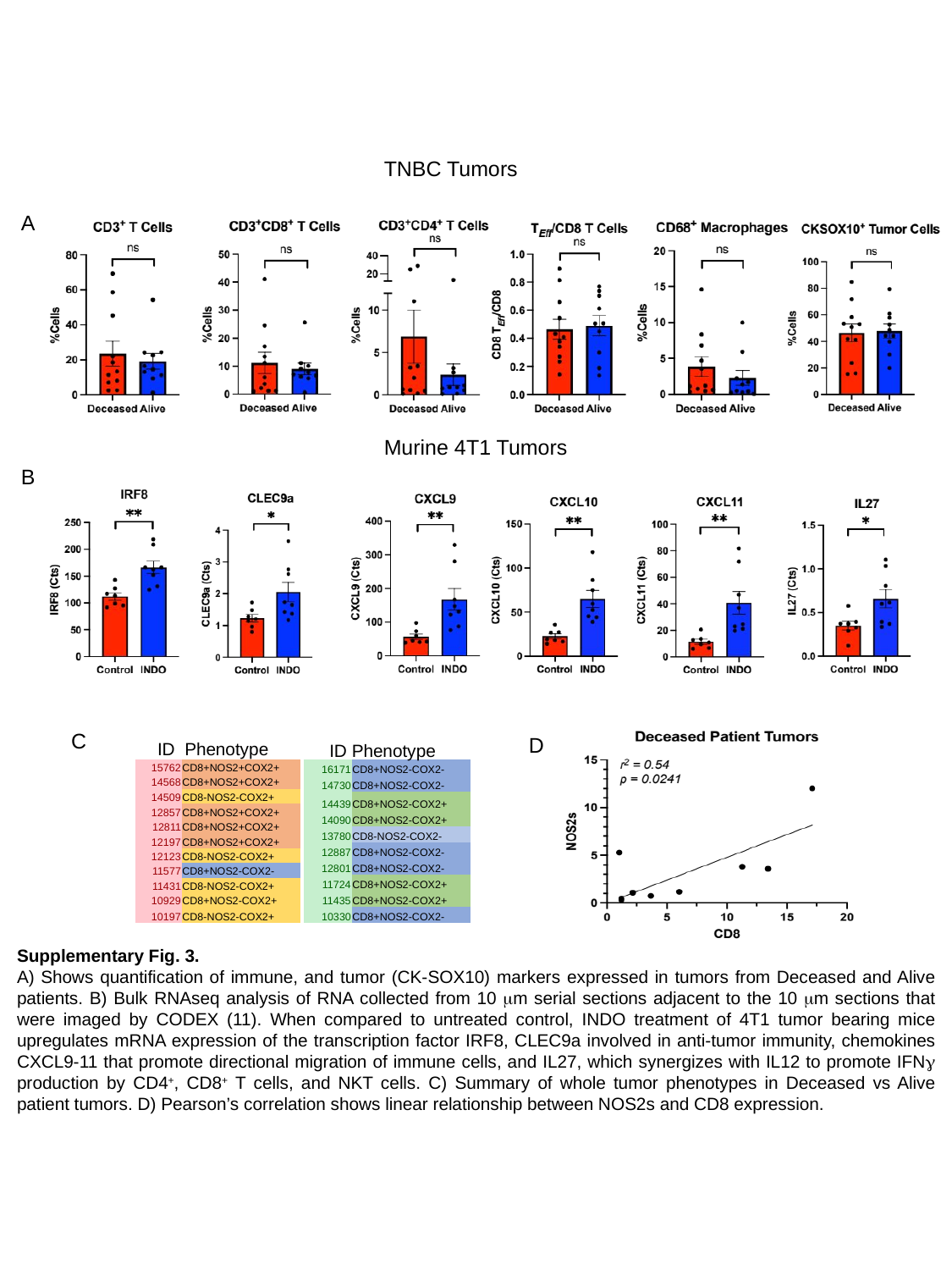

TNBC Tumors
Murine 4T1 Tumors
A
B
C
 ID Phenotype
 ID Phenotype
D
Supplementary Fig. 3.
A) Shows quantification of immune, and tumor (CK-SOX10) markers expressed in tumors from Deceased and Alive patients. B) Bulk RNAseq analysis of RNA collected from 10 mm serial sections adjacent to the 10 mm sections that were imaged by CODEX (11). When compared to untreated control, INDO treatment of 4T1 tumor bearing mice upregulates mRNA expression of the transcription factor IRF8, CLEC9a involved in anti-tumor immunity, chemokines CXCL9-11 that promote directional migration of immune cells, and IL27, which synergizes with IL12 to promote IFNg production by CD4+, CD8+ T cells, and NKT cells. C) Summary of whole tumor phenotypes in Deceased vs Alive patient tumors. D) Pearson’s correlation shows linear relationship between NOS2s and CD8 expression.
| 15762 | CD8+NOS2+COX2+ |
| --- | --- |
| 14568 | CD8+NOS2+COX2+ |
| 14509 | CD8-NOS2-COX2+ |
| 12857 | CD8+NOS2+COX2+ |
| 12811 | CD8+NOS2+COX2+ |
| 12197 | CD8+NOS2+COX2+ |
| 12123 | CD8-NOS2-COX2+ |
| 11577 | CD8+NOS2-COX2- |
| 11431 | CD8-NOS2-COX2+ |
| 10929 | CD8+NOS2-COX2+ |
| 10197 | CD8-NOS2-COX2+ |
| 16171 | CD8+NOS2-COX2- |
| --- | --- |
| 14730 | CD8+NOS2-COX2- |
| 14439 | CD8+NOS2-COX2+ |
| 14090 | CD8+NOS2-COX2+ |
| 13780 | CD8-NOS2-COX2- |
| 12887 | CD8+NOS2-COX2- |
| 12801 | CD8+NOS2-COX2- |
| 11724 | CD8+NOS2-COX2+ |
| 11435 | CD8+NOS2-COX2+ |
| 10330 | CD8+NOS2-COX2- |
