# Supplementary material for: Tumor NOS2 and COX2 Spatial Juxtaposition with CD8+ T Cells Promote Metastatic and Cancer Stem Cell Niches that Lead to Poor Outcome in ER− Breast Cancer
Source: Cancer Res Commun. 2024 Oct 23;4(10):2766–82. doi: 10.1158/2767-9764.CRC-24-0235 (PMC11497117; doi:10.1158/2767-9764.CRC-24-0235)
Supplement: Supplementary Figure 4 — Supplementary Fig. 4. Supplementary Fig. 4. Spatial UMAP analysis of CD8+/-NOS2+/-COX2+/- phenotypes in Deceased vs Alive patient tumors. Single cell neighborhood profile summary. [file crc-24-0235_supplementary_figure_4_suppsf4.pptx]

## Slide 1
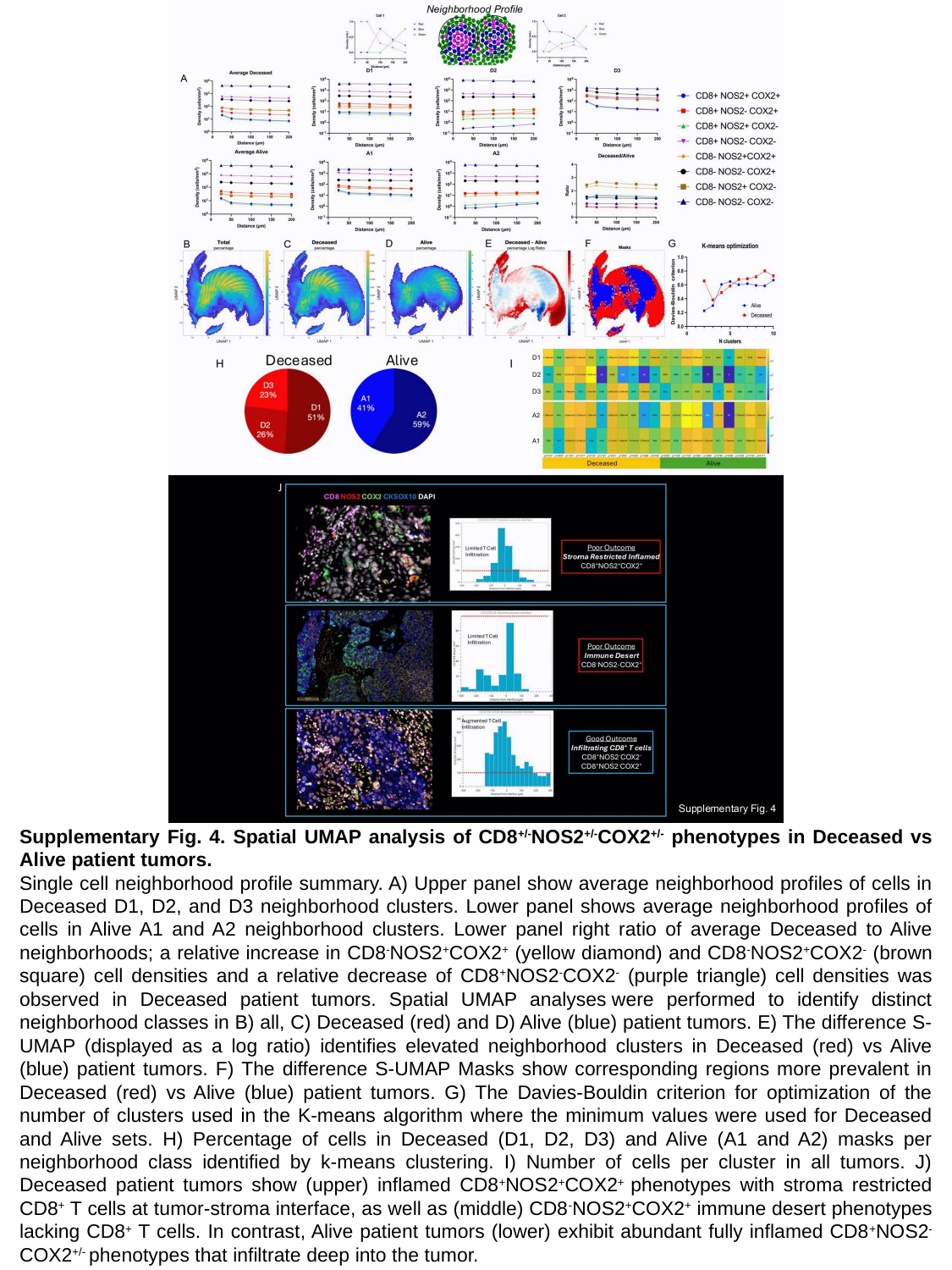

Supplementary Fig. 4. Spatial UMAP analysis of CD8+/-NOS2+/-COX2+/- phenotypes in Deceased vs Alive patient tumors.
Single cell neighborhood profile summary. A) Upper panel show average neighborhood profiles of cells in Deceased D1, D2, and D3 neighborhood clusters. Lower panel shows average neighborhood profiles of cells in Alive A1 and A2 neighborhood clusters. Lower panel right ratio of average Deceased to Alive neighborhoods; a relative increase in CD8-NOS2+COX2+ (yellow diamond) and CD8-NOS2+COX2- (brown square) cell densities and a relative decrease of CD8+NOS2-COX2- (purple triangle) cell densities was observed in Deceased patient tumors. Spatial UMAP analyses were performed to identify distinct neighborhood classes in B) all, C) Deceased (red) and D) Alive (blue) patient tumors. E) The difference S-UMAP (displayed as a log ratio) identifies elevated neighborhood clusters in Deceased (red) vs Alive (blue) patient tumors. F) The difference S-UMAP Masks show corresponding regions more prevalent in Deceased (red) vs Alive (blue) patient tumors. G) The Davies-Bouldin criterion for optimization of the number of clusters used in the K-means algorithm where the minimum values were used for Deceased and Alive sets. H) Percentage of cells in Deceased (D1, D2, D3) and Alive (A1 and A2) masks per neighborhood class identified by k-means clustering. I) Number of cells per cluster in all tumors. J) Deceased patient tumors show (upper) inflamed CD8+NOS2+COX2+ phenotypes with stroma restricted CD8+ T cells at tumor-stroma interface, as well as (middle) CD8-NOS2+COX2+ immune desert phenotypes lacking CD8+ T cells. In contrast, Alive patient tumors (lower) exhibit abundant fully inflamed CD8+NOS2-COX2+/- phenotypes that infiltrate deep into the tumor.
