# Supplementary material for: Tumor NOS2 and COX2 Spatial Juxtaposition with CD8+ T Cells Promote Metastatic and Cancer Stem Cell Niches that Lead to Poor Outcome in ER− Breast Cancer
Source: Cancer Res Commun. 2024 Oct 23;4(10):2766–82. doi: 10.1158/2767-9764.CRC-24-0235 (PMC11497117; doi:10.1158/2767-9764.CRC-24-0235)
Supplement: Supplementary Figure 5 — Spatial dot plot of CD8+/-NOS2+/-COX2+/- phenotypes in tumor from deceased patient. [file crc-24-0235_supplementary_figure_5_suppsf5.pptx]

## Slide 1
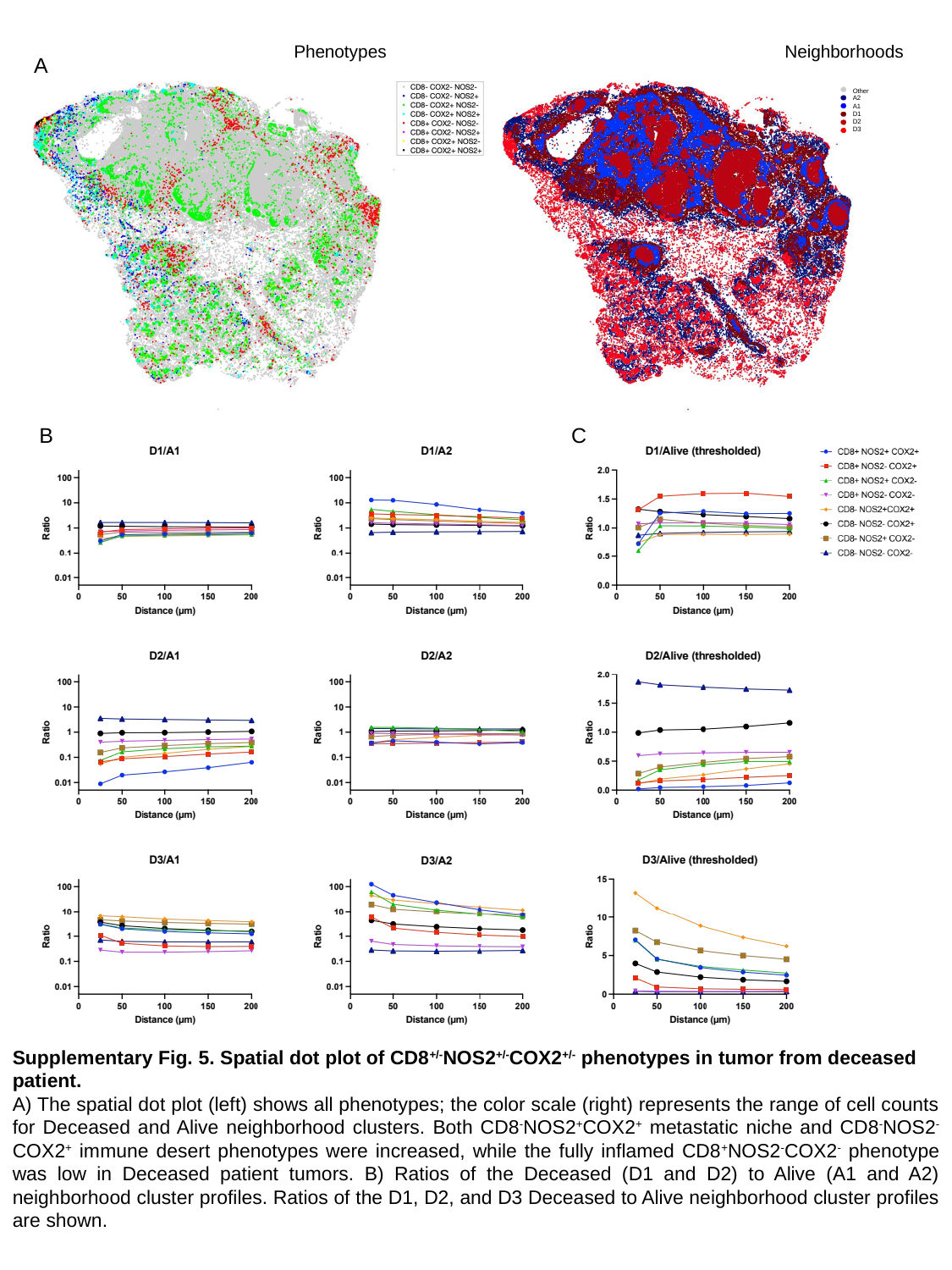

Phenotypes
Neighborhoods
A
Other
A2
A1
D1
D2
D3
B
C
Supplementary Fig. 5. Spatial dot plot of CD8+/-NOS2+/-COX2+/- phenotypes in tumor from deceased patient.
A) The spatial dot plot (left) shows all phenotypes; the color scale (right) represents the range of cell counts for Deceased and Alive neighborhood clusters. Both CD8-NOS2+COX2+ metastatic niche and CD8-NOS2-COX2+ immune desert phenotypes were increased, while the fully inflamed CD8+NOS2-COX2- phenotype was low in Deceased patient tumors. B) Ratios of the Deceased (D1 and D2) to Alive (A1 and A2) neighborhood cluster profiles. Ratios of the D1, D2, and D3 Deceased to Alive neighborhood cluster profiles are shown.
