# Supplementary material for: Tumor NOS2 and COX2 Spatial Juxtaposition with CD8+ T Cells Promote Metastatic and Cancer Stem Cell Niches that Lead to Poor Outcome in ER− Breast Cancer
Source: Cancer Res Commun. 2024 Oct 23;4(10):2766–82. doi: 10.1158/2767-9764.CRC-24-0235 (PMC11497117; doi:10.1158/2767-9764.CRC-24-0235)
Supplement: Supplementary Figure 6 — Modulation of EpCAM and CD44v6+. [file crc-24-0235_supplementary_figure_6_suppsf6.pptx]

## Slide 1
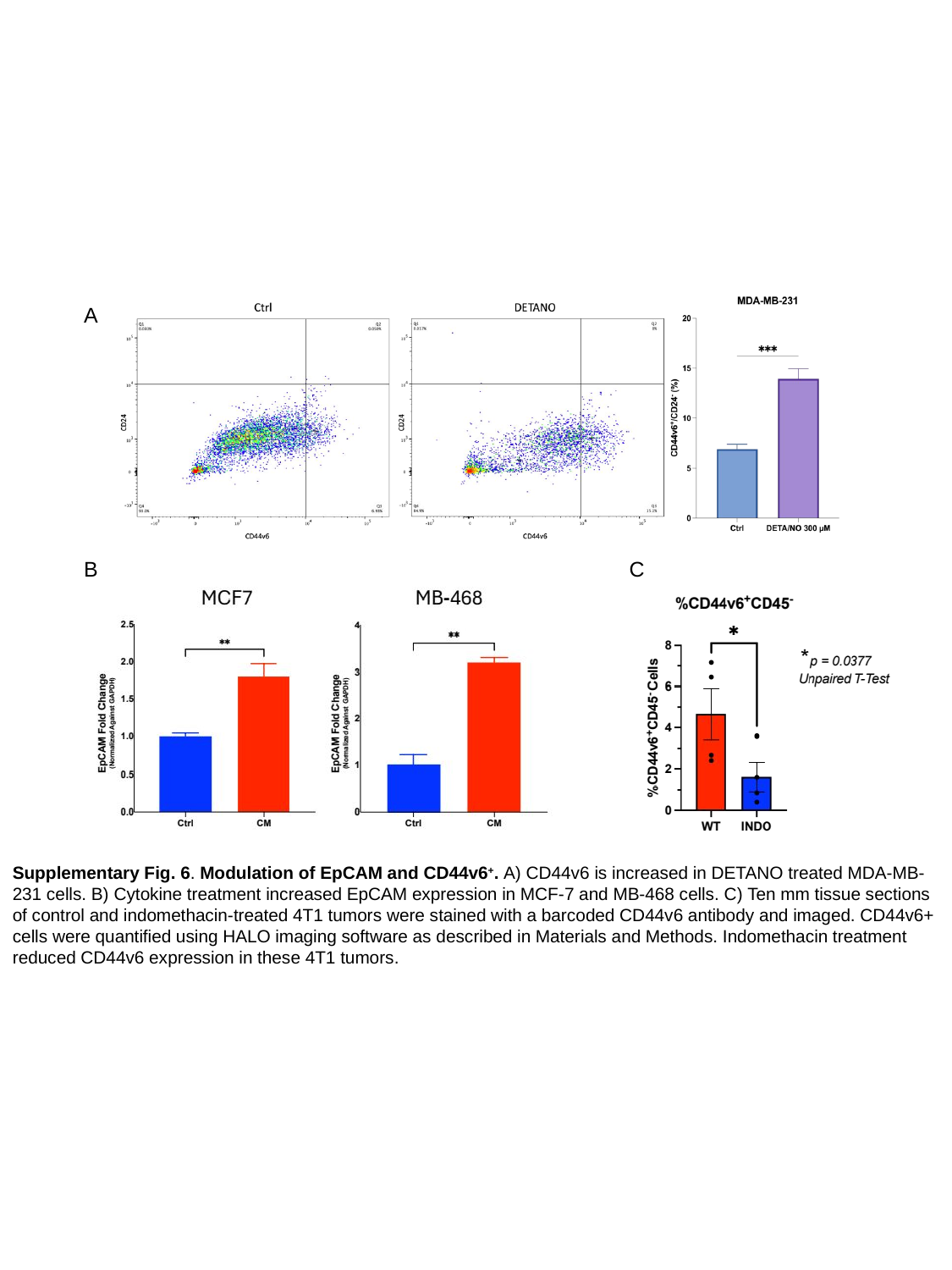

A
B C
Supplementary Fig. 6. Modulation of EpCAM and CD44v6+. A) CD44v6 is increased in DETANO treated MDA-MB-231 cells. B) Cytokine treatment increased EpCAM expression in MCF-7 and MB-468 cells. C) Ten mm tissue sections of control and indomethacin-treated 4T1 tumors were stained with a barcoded CD44v6 antibody and imaged. CD44v6+ cells were quantified using HALO imaging software as described in Materials and Methods. Indomethacin treatment reduced CD44v6 expression in these 4T1 tumors.
